# Efficacy and External Validity of Electronic and Mobile Phone-Based Interventions Promoting Vegetable Intake in Young Adults: Systematic Review and Meta-Analysis

**DOI:** 10.2196/jmir.5082

**Published:** 2016-04-08

**Authors:** Monica Nour, Juliana Chen, Margaret Allman-Farinelli

**Affiliations:** ^1^ School of Life and Environmental Sciences University of Sydney Sydney, NSW Australia

**Keywords:** young adults, vegetable consumption, mHealth, eHealth, social marketing

## Abstract

**Background:**

Young adults (18–35 years) remain among the lowest vegetable consumers in many western countries. The digital era offers opportunities to engage this age group in interventions in new and appealing ways.

**Objective:**

This systematic review evaluated the efficacy and external validity of electronic (eHealth) and mobile phone (mHealth) -based interventions that promote vegetable intake in young adults.

**Methods:**

We searched several electronic databases for studies published between 1990 and 2015, and 2 independent authors reviewed the quality and risk of bias of the eligible papers and extracted data for analyses. The primary outcome of interest was the change in vegetable intake postintervention. Where possible, we calculated effect sizes (Cohen *d* and 95% CIs) for comparison. A random effects model was applied to the data for meta-analysis. Reach and representativeness of participants, intervention implementation, and program maintenance were assessed to establish external validity. Published validation studies were consulted to determine the validity of tools used to measure intake. We applied the Grading of Recommendations Assessment, Development and Evaluation (GRADE) system to evaluate the overall quality of the body of evidence.

**Results:**

Of the 14 studies that met the selection criteria, we included 12 in the meta-analysis. In the meta-analysis, 7 studies found positive effects postintervention for fruit and vegetable intake, Cohen *d* 0.14–0.56 (pooled effect size 0.22, 95% CI 0.11–0.33, I^2^=68.5%, *P*=.002), and 4 recorded positive effects on vegetable intake alone, Cohen *d* 0.11–0.40 (pooled effect size 0.15, 95% CI 0.04–0.28, I^2^=31.4%, *P*=.2). These findings should be interpreted with caution due to variability in intervention design and outcome measures. With the majority of outcomes documented as a change in combined fruit and vegetable intake, it was difficult to determine intervention effects on vegetable consumption specifically. Measurement of intake was most commonly by self-report, with 5 studies using nonvalidated tools. Longer-term follow-up was lacking from most studies (n=12). Risk of bias was high among the included studies, and the overall body of evidence was rated as low quality. The applicability of interventions to the broader young adult community was unclear due to poor description of external validity components.

**Conclusions:**

Preliminary evidence suggests that eHealth and mHealth strategies may be effective in improving vegetable intake in young adults; whether these small effects have clinical or nutritional significance remains questionable. With studies predominantly reporting outcomes as fruit and vegetable intake combined, we suggest that interventions report vegetables separately. Furthermore, to confidently establish the efficacy of these strategies, better-quality interventions are needed for young adults, using valid measures of intake, with improved reporting on costs, sustainability and long-term effects of programs.

**Trial registration:**

PROSPERO International Prospective Register of Systematic Reviews: CRD42015017763; http://www.crd.york.ac.uk/PROSPERO/display_record.asp?ID=CRD42015017763 (Archived by WebCite at http://www.webcitation.org/6fLhMgUP4)

## Introduction

Poor fruit and vegetable intake contributes to 2.635 million deaths per year [1]. Consuming the recommended 600 g daily could reduce this global burden by 1.8% [1], with adequate fruit and vegetable intake linked to minimized adiposity, improved weight management [2], and reduced risk of heart disease and some cancers [1]. Despite several decades of government-led social marketing campaigns, alongside concerted effort by researchers and practitioners to facilitate behavior change, intake of vegetables remains suboptimal in many countries [3-6].

Australian young adults (18-34 years) are among the lowest consumers of vegetables, with only 4.7% consuming the recommended 5 or more servings a day [7]. During this transitional phase of life, young adults are developing self-determined food habits that will affect their future health. While the association between fruit and vegetable consumption and reduced chronic disease risk is well established in the literature [2,8-15], promoting these long-term health benefits, as is typically done in nationwide social marketing campaigns, does not appear to motivate young adults [16,17]. Young adults are typically less concerned about their future well-being and engage in more risky health behaviors [18]. Consequently, this population should be targeted separately in interventions.

Research in the area of digital interventions has revealed that electronic (eHealth) and mobile phone (mHealth) -based strategies are effective in promoting healthful behaviors [19-21]. eHealth and mHealth refer to the use of the Internet, mobile, or wireless devices to deliver health services and information to improve health outcomes or enhance health research [22,23]*.* Examples of eHealth and mHealth strategies include text messaging, email, mobile phone apps, phone calls, and websites. Young adults are among the highest users of mobile phones and wireless information sharing platforms [24], with 89% of 18- to 29-year-olds in the United States reporting use of social networking sites [25]. This offers an opportunity to engage young adults in interventions in new and appealing ways. Harnessing this technology to deliver social marketing and individually tailored programs could facilitate the widespread dissemination of interventions in an affordable, convenient, and age-appropriate manner.

Previous systematic reviews of fruit and vegetable consumption-promoting programs have identified that, while interventions produced some positive changes in knowledge and attitudes about the importance of fruit and vegetable consumption, there were only minor improvements in intake [26-28]. These interventions were typically delivered to adults and children, and targeted fruit and vegetable intake concurrently. To our knowledge, to date there is no published review investigating the efficacy and external validity of social marketing and eHealth and mHealth interventions on vegetable intake in young adults. With greater perceived barriers for the consumption of vegetables, poorer knowledge about vegetable servings [29], and just over half of the population already meeting the recommended 2 fruit servings a day [7], it is evident that increasing vegetable intake is a greater challenge. Thus, investigating the implications of interventions on vegetable intake alone will help us understand how we can better support and facilitate improved vegetable consumption.

When evaluating the efficacy of interventions, the accuracy of outcomes should be considered. This is dependent on the validity of intake measurement tools. To compare outcomes across studies, definitions of what constitutes a vegetable serving is also important. This is a source of confusion for the public and for researchers, with variations between countries [30]. In Australia, a serving of vegetables is approximately 75 g or half a cup of cooked vegetables [31], whereas in the United Kingdom a serving is equivalent to 80 g [32].

Furthermore, the specification of behavior change techniques used in interventions is essential to the process of revealing which strategies are effective in the target population and allowing replication of successful interventions [33]. A review of recent eHealth and mHealth interventions found that studies that incorporated a greater number of behavior change techniques had the largest effects [34]. Whether these effects can be generalized to the broader young adult population depends on external validity. Thus, evaluating the external validity of studies is as important as determining efficacy and will have implications for the translation of interventions into larger health promotion programs.

Therefore, in this review we aimed to (1) systematically examine the efficacy of social marketing, and electronic or mobile phone-based interventions in increasing vegetable intake in young adults, (2) assess the quality of the studies, including the validity of tools used to monitor changes in vegetable intake, and (3) review the adequacy of reporting of external validity components.

## Methods

We used the Preferred Reporting Items for Systematic Reviews and Meta-Analyses (PRISMA) framework [35] to develop the systematic review protocol, which has been published elsewhere [36]. During the review process, we replaced the quality-assessment tool specified in the original protocol with the Grading of Recommendations Assessment, Development and Evaluation (GRADE) system [37].

### Search Strategy

We conducted the systematic literature search between April and August 2015 using the following electronic databases: ScienceDirect, MEDLINE, PyscINFO, Scopus, the Cochrane Library, CINAHL, Embase, and Web of Science. The last search was conducted on August 17, 2015, with no new relevant papers found. We excluded studies published before 1990, as email was not widely used before this period [38]. After hand searching reference lists of key reviews and included studies, as well as conducting a manual search of JMIR journals, we included other relevant studies.

We conducted 2 searches. The first used combinations, synonyms, and truncations of “online intervention,” “computer-assisted therapy,” “electronic mail,” “Internet,” “website,” “cell phones,” “young adult” or “adult,” “fruits,” and “vegetables.” While we were searching largely for eHealth and mHealth interventions, we used other relevant MEDLINE MeSH, such as “telemedicine,” to encompass the terms “mHealth,” “eHealth,” “telehealth,” and “mobile health.” Furthermore, although we were mainly interested in the efficacy of vegetable interventions, we extended the search terms to include “fruit,” as studies typically report on fruit and vegetables concurrently. Additionally, we used the term “adult” alongside “young adult” to broaden the search from 18- to 24-year-olds (the typical database definition of young adults) to 18- to 35-year-olds (based on the US National Institutes of Health cut-off for young adults) [39]. Table 1 shows the first search strategy used in the MEDLINE. The full search strategy is presented in Multimedia Appendix 1 (Tables S1 and S2).

**Table 1:** Electronic database search: MEDLINE (search 1: eHealth and mHealth interventions).

Search number	Search statement^a^	No. of citations retrieved
1	Online intervention.mp or Computer-assisted therapy.mp. or Therapy, Computer-Assisted/	5242
2	Internet/ or Website.mp	55,352
3	Cell phones.mp or Cell phones/	5040
4	Telemedicine/ or Cyber.mp	12,148
5	email.mp or Electronic mail/	5193
6	Adult/or Young adult/ or young adult*.mp	4,093,057
7	Fruit/ or Fruit*.mp	65,586
8	Vegetable*.mp or Vegetables/	39,576
9	1 or 2 or 3 or 4 or 5	77,751
10	7 or 8	87,363
11	6 and 9 and 10	120
12	Limit 11 to (English language and humans and yr = 1990-current)	120

^a^Modifiers are * (search term as major focus of articles), .mp (multiple purpose search including all fields: title, original title, abstract, subject heading, name of substance, and registry word fields), and / (valid controlled vocabulary term which has been searched in the subject headings field of the database).

We conducted separate database and Google searches to locate programs that used social marketing and mass media to increase fruit and vegetable intake in young adults. Search terms were “young adult,” “adults,” “fruits,” “vegetables,” “social marketing,” “social media,” and “mass media.” These studies were not limited by publication type and included gray literature, such as nonpublished evaluations of programs by organizations. Table 2 presents the second search strategy used in MEDLINE.

**Table 2:** Electronic database search: MEDLINE (search 2: social marketing and mass media interventions).

Search number	Search statement^a^	No. of citations retrieved
1	Adult/ or Young Adult/ or young adult*.mp.	4126,552
2	Fruit/ or fruit*.mp.	66,529
3	Vegetable*.mp. or Vegetables/	40,014
4	2 or 3	88,502
5	Social marketing.mp. or social marketing/	2976
6	Social media. mp or Mass Media/ or Social Media/	11,192
7	5 or 6	13,882
8	1 and 4 and 7	6
9	Limit 8 to (English language and humans and yr = 1990-current)	6

^a^Modifiers are * (search term as major focus of articles), .mp (multiple purpose search including all fields: title, original title, abstract, subject heading, name of substance, and registry word fields), and / (valid controlled vocabulary term which has been searched in the subject headings field of the database).

### Eligibility Criteria

Criteria for inclusion of eHealth and mHealth interventions were as follows: (1) randomized controlled trials (RCTs) with a primary or secondary aim of increasing fruit and vegetable intake in young adults that (2) were targeted at young adults aged 18–35 years inclusive, (3) reported fruit and vegetable intake at baseline and follow-up, (4) involved healthy participants with no disease or illness that would affect the primary outcome or ability to modify fruit and vegetable intake, (5) were written in English, (6) were published after 1990, and (7) were limited to eHealth- and mHealth-based interventions, defined as studies using texting, email, mobile phone apps, phone calls, or websites to deliver the intervention.

Criteria for inclusion of social marketing and mass media interventions were identical to points (1) to (6) above, but were not limited by study design. Social marketing and mass media interventions were defined as those that used media advertising through the Internet, television, billboards, radio, or social media platforms such as Facebook.

### Study Selection

**Figure 1:**
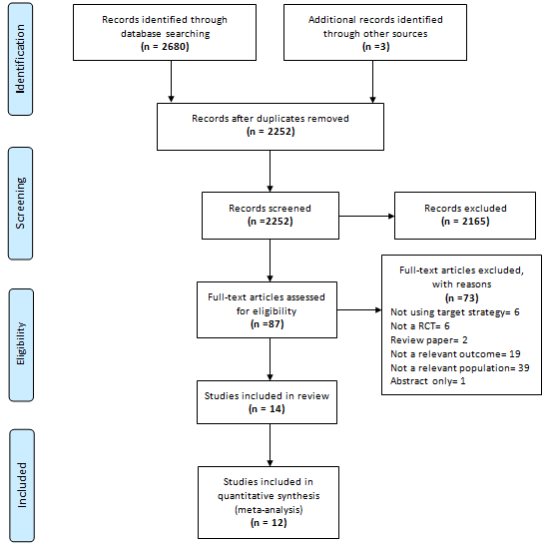
Flow diagram demonstrating the process of selecting the included studies of interventions promoting fruit and vegetable intake in young adults. Other sources included a Google search, a hand search of reference lists of relevant systematic reviews and included studies, and a manual search of JMIR journals.

We downloaded titles and abstracts of all retrieved studies to EndNote X6 citation management software (Thomson Reuters). Duplicates were removed, then titles and abstracts were reviewed by grouping papers into (1) those meeting selection criteria or (2) requiring further examination; or (3) they were excluded. Papers determined to be potentially relevant to the review were downloaded as full text and reviewed for eligibility by two assessors (MN, JC) and further categorized (Figure 1). We resolved discrepancies in assessors’ results by discussion.

### Data Extraction Process

We created a data extraction table according to the principles of the PRISMA statement for reporting systematic reviews [35], with some additional elements included for completing the Cochrane Collaboration’s risk of bias tool [40]. Once we had piloted the process on a random selection of 4 of the included studies, 2 independent reviewers extracted the following data in duplicate: study details (authors, year, country of publication, funding, and affiliations); participants (characteristics, setting, inclusion and exclusion criteria, attrition, and blinding); intervention and comparator details; duration; and the summary outcome measure (change in fruit and vegetable intake between baseline and follow-up for the intervention and control arms). We also extracted the name of the tool used to assess changes in fruit and vegetable intake, as well as citations of available validation studies.

### Data Synthesis and Analysis

The primary outcome of interest was the change in vegetable intake postintervention. Where possible, for all study arms we recorded mean or median intakes (as servings, cups, frequency, or percentage consuming) pre- and postintervention. If vegetable intake was not reported separately, we documented the change in fruit and vegetable intake. We also noted the measures of error (SE or SD) and associated *P* values for change between groups over time. To determine the magnitude of intervention outcomes, we calculated effect sizes (Cohen *d* and 95% CIs) for studies that reported sufficient data (means, and measure of error or frequencies). Web-based calculators [41] based on Lipsey and Wilson’s formulas [42] assisted with calculations. We assessed the magnitude of the effect sizes according to Cohen’s categories, whereby an effect <0.2 is considered negligible, between 0.2 and 0.49 is small, 0.5-0.8 is medium, and >0.8 is large [43].

We also considered the clinical significance of outcomes. There is no consensus in the literature regarding what change in intake is considered clinically significant. However, several meta-analyses and longitudinal studies suggest a dose-response relationship, whereby an increase in vegetable intake by approximately 1 serving is protective for cardiovascular health (decreased risk of stroke and cardiovascular disease mortality by 11% and 4%, respectively) [44,45]. Furthermore, every 1-serving increase in vegetable intake has been associated with a 0.12 kg reduction in weight (95% CI -0.35 to -0.14) [46]. These studies define a serving of vegetables as approximately 1 cup of leafy vegetables or half a cup of cooked vegetables (frozen, fresh, or canned) in line with previous US and current Australian dietary guidelines [31,47].

To pool the outcomes for the meta-analysis, we grouped studies for which an effect size was calculated. We used STATA version 13 (StataCorp LP) to conduct the analyses using the metan, metabias, and metafunnel commands. A random effects model was applied. Publication bias was determined through Egger’s statistical test for funnel plot asymmetry and visual inspection of the funnel plots of the Cohen *d* effect size (standardized mean difference), plotted against its standard error. The I^2^ value for heterogeneity was calculated based on the Q statistic: [(Q statistic - df/Q statistic) × 100%]. Cochrane Collaboration guidelines [48] suggest that an I^2^ for heterogeneity below 40% is considered low, and a value above 50% is considered substantial.

### Quality Assessment

#### Risk of Bias Assessment

Using the Cochrane Collaboration’s tool [40], we established risk of bias at the individual study level, based on the following study elements: selection of participants (random sequence generation and concealment of allocation methods); attrition (completeness of outcome data); detection (blinding of participants and personnel); and reporting (selective reporting of outcome measures). Two authors (MN and JC) independently evaluated each study for risk of bias and coded them as having low risk, high risk, or unclear risk. Any differences in judgment were clarified through discussion.

#### GRADE Assessment

The quality of the body of evidence was determined by 2 independent reviewers (MN, JC) using the GRADE system [37]. We considered 5 categories to ascribe a quality rating: limitations in study designs; consistency of results; directness of the evidence with regard to study populations, intervention design, and outcomes measured; precision of outcomes; and the presence of publication biases.

### Rating Validity of Dietary Assessment Tools

We determined the validity of each tool used to measure changes in vegetable intake based on published literature demonstrating its accuracy [49-59]. The checklist of requirements by Nelson et al [60] was also consulted to qualitatively examine the effectiveness of reporting on measurement tools. This checklist assesses factors such as data-collection procedures (objective measure vs self-report), methods of quantifying portions, variety of foods captured, food composition databases used and whether checking procedures were applied.

### Rating External Validity

We assessed the external validity of included studies based on the Green and Glasgow’s criteria [61]. The assessment explored components under 3 sections: (1) reach and representativeness of participants, (2) intervention implementation and adaptation, and (3) program maintenance and institutionalization (sustainability of program implementation). Quantitative and qualitative data pertaining to these external validity components were extracted. We recorded specific data that were not reported as not reported, and if an assessment component did not apply to the particular study we reported it not applicable. Individual participation rate (%) was calculated as the percentage of eligible participants agreeing to participate. Attrition rate (%) was calculated as the percentage of participants who dropped out after randomization. Attrition was further grouped by intervention arm (treatment vs control). Extracted data were used to examine the number of studies adhering to the external validity components. The frequency and adequacy of reporting of these components were also examined and compared between studies.

## Results

### Study Selection

As the flow diagram in Figure 1 shows, we found 2680 studies through database searching and 3 additional studies through hand searching the references. We screened a total of 2252 papers by title and abstract. Of these, we assessed the full text of 87 studies. A total of 14 studies [62-75] met the selection criteria and were included in the review. See Multimedia Appendix 2 for the complete list of references excluded by full text with corresponding reasons. None of these studies used social marketing strategies or mass media to encourage vegetable consumption in young adults specifically. Therefore, the remaining results report the effectiveness and external validity of eHealth and mHealth interventions aimed at increasing fruit and vegetable intake in young adults. We included 12 studies in the meta-analyses. For the meta-analysis, we combined the reported results in 2 groups for comparability: fruit and vegetable (8 studies) and vegetable only (5 studies); 1 study contributed results for both groups [64].

### Study Reach and Representativeness of Participants

Overall, 7984 healthy people participated in the eHealth and mHealth RCTs (see Multimedia Appendix 3, Table S3). There were, however, large discrepancies in the sample sizes. Only 3 of the 14 studies had recruited >500 participants at baseline [62-64], and 1 study had a sample size of <100 [64]. More than half of the interventions were conducted in the United States [62,64,67,69,71,73,75], 4 in Australia [65,65,67,72], 1 in New Zealand [74], and 1 in Malaysia [70]. The target audience was college or university students for the majority of the studies [62,63,67-75], and 3 studies reported their target audience to be young adults [64-66].

Recruitment methods were reported for 13 of the 14 studies, but limited details were provided. All but 2 studies recruited through the university or college setting [64,66]. Participants were recruited through undergraduate psychology courses in 2 studies [67,68], from random nonnutrition classes in 2 studies [69,70], and through advertisements and flyers posted on university grounds in 4 studies [62,63,65,71,72]. In 1 study a recruitment table was set up on campus [73], and another study invited patients attending the student university health service [74]. In 1 study [64] advertisements with a toll-free phone number were used, and the final study distributed letters of invitation through participating family doctors, along with electronic and print advertisements [66]. Of the included studies, 9 indicated their participation rate, with a mean of 78.0%. The inclusion criteria were detailed by 10 studies, all of which specified age (years) as one of their criteria. Demographic data were provided by most of the studies although not consistently. Baseline age (years) was reported in all but 1 study (Multimedia Appendix 3, Table S3), with a mean age of 20.8 years across the studies. The ethnicity of participants was reported to be >50% Caucasian or white in 7 studies. The percentage of female participants was reported by 13 studies, with women more commonly recruited than men (mean 69.8% female) (Multimedia Appendix 3, Table S3).

### Intervention Implementation and Adaptation

Details of the intervention and comparator groups were provided in detail. All studies recruited an intervention and a control group (see Multimedia Appendix 4, Table S4), with 4 studies using multiple intervention and control arms [67,71,72,74]. A total of 6 studies provided no treatment to the control arm [67,68,70,71,74,75], 7 studies gave the comparator group general information not containing the intervention material [62,64-66,69,72,73], and 1 study provided the control group with the intervention material on completion of the follow-up assessment [63]. The duration of interventions and number of sessions were easily extrapolated from each study. The level of contact between researchers and participants ranged from one-off sessions (provision of feedback) to daily contact by email or text message (Multimedia Appendix 4, Table S4). The majority of the interventions used online education through learning platforms, websites, and emailing, with only 2 studies using apps [65,66] and 4 using text messaging [65,66,70,72]. No studies reported the use of social media platforms. The studies predominantly used goal setting for behavior change, with monitoring and feedback also commonly incorporated. For the majority of the interventions, the aim was to offset weight gain in young adulthood. Targeting improvements in fruit and vegetable intake was one such method used to address weight gain. While 1 study was designed to reduce health-risk behaviors in young adults [74], only 5 studies focused specifically on fruit and vegetable intake [64,68,69,72,75], and none targeted vegetables alone.

The reviewed studies varied in the detail provided regarding the behavior theories and techniques considered in the intervention design. The design of 5 studies was based on the transtheoretical model of behavior change, where the participants’ stage of change determined the content received [63-66,75]. A total of 6 studies were theory or education based [62,63,67,70,71,73]. Social-cognitive theory informed 2 interventions [67,69]*.* Half of the reviewed studies applied the behavioral construct of self-efficacy in their intervention [62,64,69,70,71,73,75]. The study by Kypri and McAnally [74] did not report consideration of theoretical frameworks in their intervention design. The remaining 2 studies [68,72] were informed by the theory of planned behavior and the theory of habit formation (Multimedia Appendix 4, Table S4). All the studies that we reviewed intervened at the individual level. Only 2 studies were implemented outside of the university setting, thus limiting the generalizability of the interventions to the overall young adult population. Of these studies, one [64] was targeted at lower socioeconomic status young adults, while the other mainly captured young adults from higher socioeconomic areas [66].

The duration of the interventions (excluding postintervention follow-up) ranged from one-off contact to 6 months of treatment, with a mean of 10 weeks (Multimedia Appendix 4, Table S4). A total of 9 studies allocated a follow-up period [62-64,66,69,71-73], with a mean of 16 weeks. Adherence was most commonly documented as the number of sessions completed or the amount of materials viewed by participants (Multimedia Appendix 4, Table S4), but was not consistently reported across studies. The mean level of compliance among those reporting adherence was 85.4%.

Delivery expertise varied among the studies (Multimedia Appendix 4, Table S4). Research staff were more commonly reported to have conducted the interventions, with little specification of their qualifications and the number of research staff involved. Registered dietitians delivered 5 of the interventions [63,65,66,69,75]. Other expertise included a health promotion officer [71] and outreach educators [64].

### Study Maintenance and Institutionalization

The rate of attrition was documented in all reviewed studies. At completion of the interventions the mean attrition rate was 19.6% (see Multimedia Appendix 5, Table S5). All but 4 studies [64,69,71,75] reported attrition for the control and intervention group separately, and 4 did not assess differences in characteristics between completers and noncompleters [65,70,71,73]. Only 2 studies looked at the long-term impacts of the study, by assessing outcomes at least 12 months following treatment [62,63]. Both of these studies found that the changes in fruit and vegetable intake were not maintained at follow-up (Multimedia Appendix 5, Table S5). The sustainability of program implementation was poorly reported, with only 1 study mentioning that results would be used to refine the intervention for trial in a broader young adult population using a larger sample size [66]. Finally, only 2 studies published a process evaluation documenting effective program elements [62,66].

### Risk of Bias

We rated the majority of the studies reviewed as unclear to high risk because they did not perform intention-to-treat analyses, which introduced biases in the outcome data (attrition bias) [62,70-75] (see Multimedia Appendix 6, Table S6). We rated 2 studies high in a second domain (detection bias) [71,73]. The majority of the studies did not clarify their methods of blinding (n=8). Selection bias was mainly unclear within and across studies, with 5 studies not reporting the method of sequence generation in randomization [62,64,69,71,75] and only 2 studies specifying allocation concealment methods [66,74] (Multimedia Appendix 6, Table S6). While all of the studies reported results for prespecified outcomes, we could not completely rule out reporter bias across studies because only 5 RCTs published their original protocol [63,65,66,68,69] or provided details of their trial registration [66]. However, no selective reporting was apparent based on the methods within the reviewed manuscripts (both successful and unsuccessful outcomes recorded). Overall, the combined lack of clarity of the level of bias across studies raises concerns about the plausibility of the studies’ results.

### GRADE Quality Rating

The reviewed interventions had several limitations in study design and did not address the research question directly, resulting in an overall low quality rating (Table 3).

**Table 3:** Overall assessment of quality in 14 studies (7984 participants in total) of promotion of fruit and vegetable intake using the Grading of Recommendations Assessment, Development and Evaluation (GRADE) system.

Category	Rating with reasoning
Limitations	–2 quality levels due to very serious limitations
Consistency	No subtraction of levels, as inconsistency doesn’t affect confidence in results
Directness	–2 quality levels, as the population, outcomes, and study design are indirect
Precision	No subtraction of levels due to good precision
Publication bias	No subtraction of levels, as funnel plot symmetry suggests publication bias is unlikely
Overall quality	Low: our confidence in the effect estimate is limited

#### Study Limitations

All the included studies were RCTs. However, only 2 studies adequately concealed the difference between intervention arms [66,74]. In 1 study, the study design and purpose of randomization was explained to participants, preventing allocation concealment [71]. The remaining 11 studies did not clearly describe their method of concealment. Furthermore, 8 studies did not describe their method of blinding and 3 did not blind effectively [65,71,73]. Half of the included studies had a loss to follow-up of >20% [62-64,67,72,73,75] and did not conduct intention-to-treat analysis [62,70-74]. A total of 3 studies did not state methods for dealing with missing data or conducted analysis on completer populations [63,68,69]. Several studies used nonvalidated measures of intake, further limiting the quality of the body of evidence.

#### Consistency

**Figure 2:**
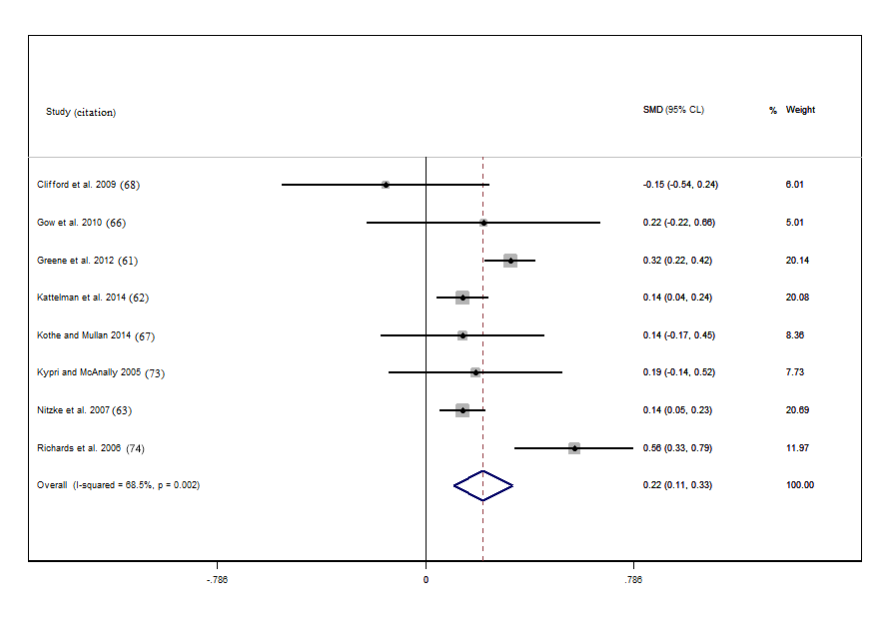
Forest plot of Cohen d effect size (standardized mean difference, SMD) for studies reporting change in fruit and vegetable intake combined. The diamond represents the overall effect size; the percentage weighting of each study toward the overall effect is indicated by the size of gray squares; and the 95% confidence limits are shown by horizontal lines. The overall intervention effect lies at the center of the larger clear diamond with right and left end points indicating the 95% confidence limits. Note: weights are from random effects analysis.

The studies with effect sizes for change in fruit and vegetable intake yielded an I^2^ statistic of 68.5% (P value for heterogeneity =.002), indicating that there may be considerable heterogeneity. However, a higher heterogeneity can be caused by small variations in point estimates from studies with larger sample sizes, as is evident in Figure 2. An I^2^ of 31.4% (P value for heterogeneity =0.2) for studies reporting vegetable intake separately suggests low heterogeneity.

#### Directness

While comparisons between control and intervention arms were direct for the included interventions, variations in study design, populations, and outcome measures meant that the overall body of evidence was indirect. The population of included studies was predominantly college students. Only 2 interventions recruited beyond the university or college setting, but they were still not representative of the broader young adult population. This review allowed for the inclusion of studies that measured changes in intake as a secondary outcome. Consequently, several studies were weight management interventions targeting fruit and vegetable intake as a component of the program. Only 5 studies targeted fruit and vegetables specifically [64,68,69,72,75] and none targeted vegetables alone. Measures of fruit and vegetable intake also varied considerably. Thus, the overall evidence is an indirect representation of the impact of eHealth and mHealth on vegetable intake.

#### Precision

Only 6 of the 14 studies reported conducting power calculations [63,67-69,71,73]. However, these were mainly based on primary outcomes other than vegetable intake, such as change in nutrition knowledge or weight. Sample size varied from 51 to 2024 participants but yielded 7984 in total, which is considered sufficient.

#### Publication Bias

**Figure 3:**
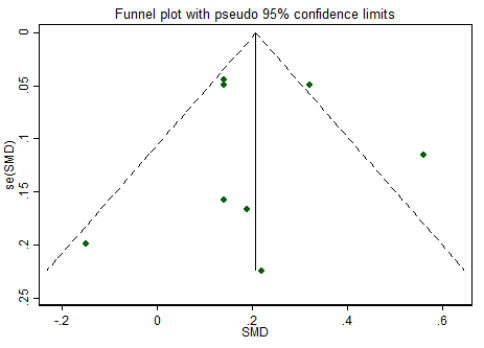
Funnel plot for risk of publication bias: intervention effect for fruit and vegetable intake represented by the standardized mean difference (SMD) plotted against the standard error, se(SMD). Dashed diagonal lines indicate the pseudo 95% confidence limits and scatter dots represent individual studies.

**Figure 4:**
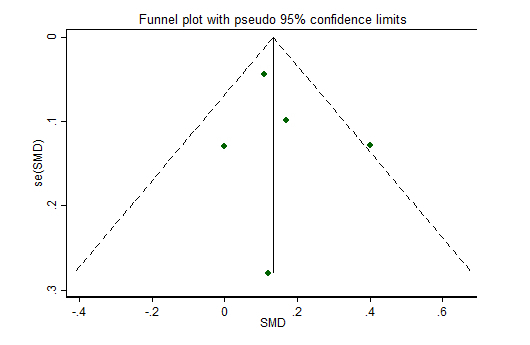
Funnel plot for risk of publication bias: intervention effect for vegetable intake represented by standardized mean difference (SMD) plotted against the standard error, se(SMD). Dashed diagonal lines indicate the pseudo 95% confidence limits and scatter dots represent individual studies.

While we implemented a comprehensive search strategy to capture the gray literature, we may have missed unpublished studies (interventions with insignificant or negative findings) or those published in journals not indexed in major databases. The outcomes of statistical tests of publication bias (Egger’s test) were not reported, as these results are less accurate when based on fewer than 10 studies or when there is significant heterogeneity [48]. Visual inspection of funnel plots (Figures 3 and 4) indicated symmetry in the distribution of points around the mean effect size, suggesting that bias from missing studies is unlikely.

### Efficacy of Interventions

Of the 14 reviewed studies, 9 provided results for fruit and vegetable intake, and we included 8 in the meta-analysis. Of these studies, 7 found positive effects postintervention [62-64,67,68,74,75] (Cohen *d* 0.14-0.56), 4 of which were statistically significant [62-64,75]. For all but 1 study [75], the magnitude of effect was small. In total, 2 studies also reported clinically significant improvements of ≥1 serving/day [62,75] (see Multimedia Appendix 7, Table S7). The pooled effect size for interventions reporting change in fruit and vegetable intake was 0.22 (95% CI 0.11 to 0.33), indicating a small positive effect of eHealth and mHealth interventions on fruit and vegetable intake. The 4 studies [62-64,75] with significant effects contributed 72.9% weighting (Figure 4). The I^2^ was 68.5%, *P*=.002, suggesting considerable heterogeneity between these studies, and so findings should be interpreted with caution.

**Figure 5:**
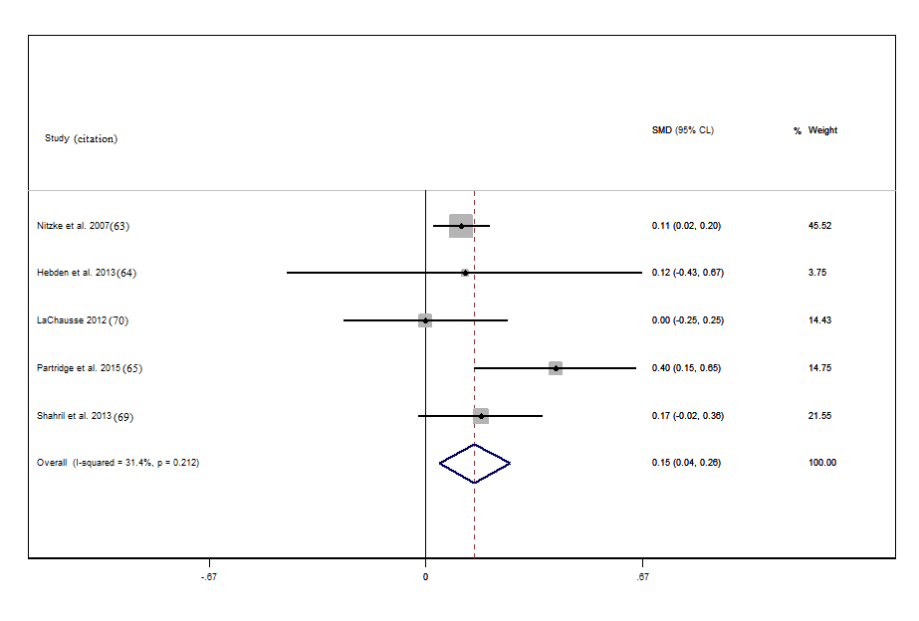
Forest plot of Cohen d effect size (standardized mean difference, SMD) for studies reporting change in vegetable intake separately. The diamond represents the overall effect size; the percentage weighting of each study toward the overall effect is indicated by the size of gray squares; and the 95% confidence limits are shown by horizontal lines. The overall intervention effect lies at the center of the larger clear diamond with right and left end points indicating the 95% confidence limits. Note: weights are from random effects analysis.

Of the 6 studies that assessed vegetable intake independently of fruit [64-66,70-72], we included 5 in the meta-analysis, 4 of which had positive effects on vegetable intake [64-66,70] (Cohen *d* 0.11-0.40). Two of these positive effects were statistically significant [64,66]. Increases in intake were <1 serving/day, with the exception of the results reported by Partridge et al [66] (Multimedia Appendix 7, Table S7). The pooled effect size for change in vegetable intake was negligible at 0.15 (95% CI 0.04 to 0.28; I^2^=31.4%, *P*=.2) (Figure 5).

Studies that were more successful in improving fruit or vegetable intake provided participants with individually tailored advice and feedback based on their stage of change [64,66,75] and incorporated goal setting [62,66,75]. Of the studies producing clinically and statistically significant results for fruit or vegetable intake, or both [62,66,75], 1 used online theory education based on nondiet principles [62]. This intervention was designed according to 2 educational models, Carey and colleague’s system of instructional design [76] and Keller’s instructional motivational model [77]. Fruit and vegetable intake goals were set after completion of each weekly educational lesson, and self-evaluation of progress preceded the next weekly Web-based module. The study by Richards and colleagues [75] used motivational interviewing in combination with Web-based resources and emails. The resources were tailored to the participants’ stage of change, where precontemplators and contemplators were given reasons to and tips on how to eat more fruits and vegetables, as well as a goal-setting framework. Action and maintenance participants received emails with tips for maintaining consumption and trying new fruits and vegetables. Finally, the study by Partridge et al [66] combined multiple eHealth and mHealth strategies to support behavior change, with text messaging found to be the most popular, and the website and discussion boards the least popular, among participants. The text messages contained reminders and tips on how to achieve their individualized goal set during their phone counseling session with a dietitian and were based on the 10 processes of change (transtheoretical model). Participants could monitor their fruit and vegetable intake goals using a personalized app that also provided recipes and tips on how to increase their intake.

### Validity of Dietary Assessment Tools

Of the reviewed studies, 5 used tools that had not been validated to assess changes in vegetable intake [68,69,71,73,75] (Table 4). While the majority of the tools were validated, only 1 was tested specifically in the young adult population [30]. Of the studies that used validated tools, short screeners were most popular, including the US National Cancer Institute’s fruit and vegetable screener [53], as well as short questions adapted from the Australian and New Zealand national nutrition surveys [52,54,56]. Furthermore, only 2 studies defined what they classified as a serving [65,66], and the outcome measure for intake lacked consistency, with studies reporting change in terms of frequency, servings or cups of vegetables consumed, as well as the percentage meeting recommendations. No studies detailed which food composition databases they used for the analysis, or whether they checked records with respondents as per the requirements specified in the Nelson and colleagues’ checklist [60]. All but 1 study [70] used a self-report measurement tool. The study by Gow and Colleagues [67] did not specify what the outcome measure was (servings vs score).

**Table 4:** Validity of tools used to measure fruit and vegetable intake and source of tools.

Author [citation]	Fruit and vegetable intake measurement tool and source [citation]	Tool validated for fruit and vegetables
Clifford et al [69]	Food frequency questionnaire adapted from US National Cancer Institute’s health habits and history questionnaire [59]	No
Franko et al [73]	Single-item question measuring daily fruit and vegetable consumption [51]	No
Gow et al [67]	Block food screener [49]	Yes
Greene et al [62]	2-item screener and National Cancer Institute screener [53]	Yes
Hebden et al [65]	Web-based short survey using questions from Australian national survey [30,52,56]	Yes
Kattelmann et al [63]	National Cancer Institute’s vegetable screener [43]	Yes
Kothe and Mullan [68]	Self-report measure of previous day’s consumption	No
Kypri and McAnally [74]	2 questions from New Zealand National Survey questionnaire [54]	Yes
LaChausse [71]	US Centers for Disease Control and Prevention’s youth risk behavior survey [58]	No
Nitzke et al [64]	5 A Day screener (7-item fruit and vegetable screener) from 5 A Day program [53]	Yes
Partridge et al [66]	Short questions adapted from the Australian National Nutrition Survey [30,52,56]	Yes
Richards et al [75]	1-item food frequency questionnaire [50]	No
Rompotis et al [72]	Short question on fruit and vegetable intake [57]	Yes
Shahril et al [70]	Diet history	NA^a^

^a^NA: Not applicable.

## Discussion

This systematic review found preliminary evidence to suggest that eHealth and mHealth interventions may have a positive impact on fruit and vegetable intake among young adults. Meta-analyses revealed a small magnitude of effect on fruit and vegetable intake and a negligible effect on vegetable intake alone. Whether these effects have clinical or nutritional significance remains questionable. The quality of the body of evidence was rated low and therefore, findings should be interpreted with caution. Rather than making recommendations, we propose suggestions for improved research.

Among the studies that improved intake, only small changes were observed (<1 serving/day). This is consistent with conclusions from existing reviews, in which interventions appear to produce minor improvements in fruit and vegetable intake [26-28]. The effectiveness of the reviewed interventions in creating sustainable change in the long term remains unclear, as follow-up periods were short. The observed dose-response clinical outcomes associated with increasing vegetable intake [44-46] are likely to become evident only in the longer term. Additionally, the link between vegetable intake and weight maintenance during the transition to adulthood occurs over time [78]. Thus, investigators should integrate longer follow-up in intervention protocols. Future studies may also consider measuring secondary outcomes, such as weight and indicators of cardiovascular health, over time to understand the longer-term clinical implications of improved vegetable intake.

With the measurement and reporting of fruit and vegetable intakes as a summed value in most studies reviewed, the impact of the eHealth and mHealth strategies on vegetable consumption specifically remains unclear. Previous research has shown that knowledge of serving sizes is poorer for vegetables than for fruit [29], and for young adults, taste was a more important barrier to increasing vegetable consumption than it was for fruit [79]. Fruit and vegetables also have varying nutrient profiles and product attributes. Considering these factors, it is apparent that vegetables should be promoted and measured separately from fruit. Additionally, most of the reviewed studies targeted fruit and vegetable intake as part of a larger weight management program. Thus, the impact of an intervention focusing primarily on vegetables is an important question for future research.

Previous research established the importance of considering behavior change theory in intervention design [33,80]. The value of incorporating behavior change theory is reiterated by this review, where the majority of the successful studies incorporated behavior change constructs such as goal setting [62,66,73,75] and the provision of individually tailored advice and feedback was based on participants’ stage of change [64,66,75]. While the transtheoretical model has been long established as an effective means of improving fruit and vegetable intake [81], these studies suggest its efficacy in eHealth and mHealth interventions where, for instance, motivational and confidence-enhancing text messages or phone calls can benefit individuals who are in the earlier contemplative stages of change. There was no clear pattern, however, to indicate that the incorporation of more behavior change techniques initiated larger improvements as previously suggested in the literature [34]. Researchers could consider investigating whether a combination of efficacious strategies and repeat exposure at a later date produces greater change to shed light on whether intensive short-duration or less-intensive, longer-duration interventions are more effective.

The mode of intervention delivery varied considerably between studies, making it difficult to determine which eHealth and mHealth strategies were most successful in supporting behavior change. However, 2 of the effective studies [66,75] used motivational phone counseling as part of their intervention. While details of the cost effectiveness of this design were not provided, generally, the individualized nature of this approach can be expensive, due to the necessity for trained staff and the monetary reimbursement required for their time. Consequently, the applicability of these studies to the whole population level may be limited. The use of other low-cost and convenient eHealth and mHealth techniques (texting and email) that can incorporate individually tailored information may be more feasible for interventions. Preliminary evidence suggests that these methods are successful [66,75]; however, further research is required to confidently determine their efficacy.

Our review was unable to identify social marketing campaigns targeted specifically at young adults. Addressing this gap is an opportunity for future public health promotion projects, with research indicating that young adults have poor awareness of population-wide campaigns and perceive considerable barriers to increasing their intake despite the promoted health benefits [82]. Additionally, we found no studies that incorporated social media platforms in their intervention. Using these high-reach and lower-cost information-sharing platforms can help to increase interactivity and collaborative content sharing. This may be the fastest and most wide-reaching way to engage young people, with approximately 89% of young adults using social media [19]. Effectiveness studies on the use of social media to improve health behaviors are limited, although preliminary reports are encouraging [83,84].

There is considerable uncertainty regarding the accuracy of the findings summarized by this review, due to the use of non-validated self-report measures of intake, which may not be sensitive enough to detect small changes and may be subject to reporter bias. Therefore, further effort is required to develop validated tools for the measurement of vegetable intake in young adults for consistent and accurate reporting of intervention outcomes. Researchers need to specify what is considered a serving of vegetables to allow easier comparison of outcomes and should use objective measures of intake for validation. Biomarkers such as vitamin C and beta-carotene are useful indicators of fruit and vegetable intake, respectively. While tests for these biomarkers are potentially costly for use in large interventions, they would be feasible and reliable in small validation studies [85].

The degree to which the interventions can be translated to the general young adult populations is questionable, as the majority of studies were conducted in the university or college setting in a sample of educated young adults. While the latest statistics indicate that an increasing proportion of young adults are enrolled in tertiary education [86], those of lower socioeconomic status remain underrepresented [87]. Future studies should limit the use of convenience sampling and aim to recruit a wider range of socioeconomic groups. Overall, the studies we reviewed did not consistently report on external validity, particularly program sustainability, costs, and long-term effects of the intervention. Process evaluations were also lacking. Consequently, the external validity of interventions for improving vegetable consumption in young adults is uncertain. There is a growing body of evidence in health research indicating that investigators are not reporting on external validity [88-90]. Improvements in this area are required to determine the potential for implementation of study designs in broader health promotion programs. Of particular importance is consideration and reporting of the costs involved in upscaling these interventions, which will have implications for health promotion officers and policy makers [91]. Furthermore, researchers should invest in conducting process evaluations to determine how to improve the efficacy of interventions and enhance their generalizability [92].

### Strengths and Limitations

This is the first systematic review to report on the effect of eHealth and mHealth interventions on vegetable intake specifically and highlights relevant opportunities for future research. We conducted the review protocol in line with the PRISMA guidelines [35] and used a comprehensive search strategy. While we searched several electronic databases and made an effort to include gray literature, we may have missed some studies. The variability across interventions with differences in study designs and measures of vegetable intake, and the overall poor study quality, made it difficult to establish definitive conclusions. Consequently, we were reluctant to rule out any eHealth or mHealth approach as ineffective and rather discussed the outcomes as a means of highlighting gaps in the current literature and opportunities for future research to generate a stronger body of evidence on whether technology-based strategies are effective in this population. Finally, the lack of consistent reporting of external validity components prevented us from making conclusions about the potential for translating interventions to the wider young adult population.

### Conclusions

Overall, this review revealed that young adults have been neglected in fruit and vegetable social marketing campaigns, and most interventions target fruit and vegetables concurrently. Very few good-quality eHealth and mHealth interventions using validated dietary assessment tools have been designed to support young adults in improving their vegetable intake. With preliminary evidence suggesting that eHealth and mHealth strategies may be an effective mode of delivering vegetable interventions, continued research using stronger and higher-quality study designs is required to better determine the efficacy of technology-based strategies for improving vegetable consumption in young adults. With previous research suggesting that multiple behavior change strategies should be used for greater improvements, researchers could consider combining promising strategies such as goal setting and tailored feedback in future interventions. The potential impact of using social media platforms to create awareness of the importance of eating enough vegetables also deserves attention. Finally, in light of the lack of reporting of external validity components in the reviewed papers, it is critical that future studies address key factors such as program costs, sustainability, and longer-term impact in order to determine the potential for upscaling interventions to the broader young adult population.

## Appendix

Multimedia Appendix 1 Additional supporting information Table s1. Search 1: e- and m-health interventions, databases searched, search terms, limits applied and results and Table s2. Search 2: social marketing and mass media interventions, databases searched, search terms, limits applied and results.

Multimedia Appendix 2 List of references excluded by full-text with reasons (n=73).

Multimedia Appendix 3 Table S3. Study descriptions of reach and representativeness of participants (n=14).

Multimedia Appendix 4 Table S4.Study Description of intervention implementation and adaption (n=14).

Multimedia Appendix 5 Table S5.Study maintenance and institutionalization (n=14).

Multimedia Appendix 6 Table S6. Risk of bias as assessed by the cochrane collaboration tool for included studies.

Multimedia Appendix 7 Table S7. Change in fruit and vegetable intake between baseline and follow-up for intervention and control arms with calculated effect size Cohen's d (95% CI) (n=14).

## Abbreviations

GRADE: Grading of Recommendations Assessment, Development and Evaluation
PRISMA: Preferred Reporting Items for Systematic Reviews and Meta-Analyses
RCT: randomized controlled trial
