# Supplementary material for: Efficacy and External Validity of Electronic and Mobile Phone-Based Interventions Promoting Vegetable Intake in Young Adults: Systematic Review and Meta-Analysis
Source: J Med Internet Res. 2016 Apr 8;18(4):e58. doi: 10.2196/jmir.5082 (PMC4841894; doi:10.2196/jmir.5082)
Supplement: Multimedia Appendix 1 [file jmir_v18i4e58_app1.pdf]

Systematic Review Search Strategy

Total search: 2680 articles (Search 1: 1482, Search 2: 1198)

Table S1. Search 1: e- and m-health interventions, databases searched, search terms, limits applied and results

Database	Search ID number	Search terms	Results
**Medline via Ovid**	1	Online intervention.mp. or Computer-assisted therapy.mp. or Therapy, Computer-Assisted/	5242
	2	Internet/ or Website.mp	55352
	3	Cell phones.mp or Cell phones/	5040
	4	Telemedicine/ or Cyber.mp	12148
	5	email.mp or Electronic mail/	5193
	6	Adult/or Young adult/ or young adult*.mp	4093057
	7	Fruit/ or Fruit*.mp	65586
	8	Vegetable*.mp or Vegetables/	39576
	9	1 or 2 or 3 or 4 or 5	77751
	10	7 or 8	87363
	11	6 and 9 and 10	120
	12	Limit 11 to (English language and humans and yr = 1990-current)	**120**
**Cochrane database of systematic reviews**	1	Online intervention.mp.	2
	2	Computer-assisted therapy.mp.	8
	3	Therapy, Computer-Assisted.mp. [mp=title, short title, abstract, full text, keywords, caption text]	30
	4	Telemedicine.mp. [mp=title, short title, abstract, full text, keywords, caption text]	52
	5	email.mp. [mp=title, short title, abstract, full text, keywords, caption text]	987
	6	cell phone.mp. [mp=title, short title, abstract, full text, keywords, caption text]	19

	7	1 or 2 or 3 or 4 or 5 or 6	1056
	8	Fruit*.mp. [mp=title, short title, abstract, full text, keywords, caption text]	243
	9	vegetable*.mp. [mp=title, short title, abstract, full text, keywords, caption text]	197
	10	8 or 9	310
	11	Adult*.mp. [mp=title, short title, abstract, full text, keywords, caption text]	5254
	12	young adult*.mp. [mp=title, short title, abstract, full text, keywords, caption text]	385
	13	11 or 12	5132
	14	7 and 10 and 13	**23**
**Web of science**	1	TS=("online intervention*" OR "computer-tailored intervention*" OR internet OR "smart-phone*" OR website* OR email OR "electronic mail")	415,764
	2	TS=(Fruit* OR vegetable*)	1,247,542
	3	TS= (“young adult*” OR Adult*)	7,121, 146
	4	Combine 1 AND 2 AND 3 *Timespan=1990-2015, search language=English*	**286**
**Science direct**	1	pub-date > 1989 and "online intervention*" OR "computer-tailored intervention*" OR internet OR "smart-phone*" OR website* OR email OR "electronic mail" AND Fruit* OR vegetable* AND “young adult*” OR Adult* AND "Randomised controlled trial".	**675**
**Cinahl**	1	"online intervention"	35
	2	(MH "Therapy, Computer Assisted+")	3,232

	3	(MM "Internet") OR "internet"	32,763
	4	(MH "Cellular Phone+") OR "mobile phone*	5,066
	5	"email"	995
	6	(MH "Telemedicine+")	3,871
	7	(MH "Adult+") OR (MH "Young Adult")	682,569
	8	(MH "Fruit+") OR "fruit"	13,277
	9	(MH "Vegetables+") OR "vegetable"	11,752
	10	8 OR 9	15,668
	11	1 OR 2 OR 3 OR 4 OR 5 OR 6	41,390
	12	7 AND 10 AND 11	**26**
**Scopus**	1	"online intervention*" OR "computer-tailored intervention*" OR internet OR "smart-phone*" OR website* OR email OR "electronic mail" AND Fruit* OR vegetable* AND “young adult*” OR Adult*	**194**
	2	'computer-tailored intervention'	38
	3	‘internet’	100,043
	4	‘smartphone’	2,126
	5	‘website’	14,431
	6	‘email’	42,976
	7	Fruit or vegetable	162,239
	8	'young adult' OR adult	5,148,352
	9	1 or 2 or 3 or 4 or 5 or 6	158,507
	10	7 and 8 and 9	**156**

**PsycInfo**	1	Computer-assisted therapy.mp. or Therapy, Computer-Assisted/	598
	2	Internet/ or Website.mp	26124
	3	Cell phones.mp or Cell phones/	2536
	4	Telemedicine/ or Cyber.mp	3912
	5	email.mp or Electronic mail/	6601
	6	Adult/or Young adult/ or young adult*.mp	33446
	7	Fruit/ or Fruit*.mp	155564
	8	Vegetable*.mp or Vegetables/	3657
	9	1 or 2 or 3 or 4 or 5	35816
	10	7 or 8	16452
	11	6 and 9 and 10	2
	12	Limit 11 to (English language and humans and yr = 1990-current)	**2**

Table S2. Search 2: social marketing and mass media interventions), databases searched, search terms, limits applied and results

Database	Search ID number	Search terms	Results
**Medline via Ovid**	1	Adult/ or Young Adult/ or young adult*.mp.	4126552
	2	Fruit/ or fruit*.mp.	66529
	3	Vegetable*.mp. or Vegetables/	40014
	4	2 or 3	88502
	5	Social marketing.mp. or social marketing/	2976
	6	Social media. mp or Mass Media/ or Social Media/	11192
	7	5 or 6	13882
	8	1 and 4 and 7	6
	9	Limit 8 to (English language and humans and yr = 1990-current)	**6**
**Cochrane Database of Systematic Reviews**	1	Adult/ or Young Adult/ or young adult*.mp.	386
	2	Fruit/ or fruit*.mp.	249
	3	Vegetable*.mp. or Vegetables/	197
	4	2 or 3	294
	5	Social marketing.mp. or social marketing/	36
	6	Social media. mp or Mass Media/ or Social Media/	17
	7	5 or 6	53
	8	1 and 4 and 7	0
	9	Limit 8 to (English language and humans and yr = 1990-current)	**0**

**Web of science**	1	TS= (social media OR social marketing OR mass media)	431,098
	2	TS= (Fruit* OR vegetable*)	1,247,569
	3	TS=(“young adult*” OR Adult*)	7,123, 146
	4	1 AND 2 AND 3	**432**
**Science direct**	1	"social media" OR "social marketing" OR "mass media" AND Fruit* OR vegetable* AND "young adult*" .	**302**
**Cinahl**	1	(MH "Social Media") OR "social media"	3512
	2	(MH "Communications Media+") OR "mass media"	317,892
	3	(MH "Social Marketing") OR "social marketing"	974
	4	(MH "Adult+") OR (MH "Young Adult")	682,594
	5	(MH "Fruit+") OR "fruit"	13,283
	6	(MH "Vegetables+") OR "vegetable"	11,762
	7	1 OR 2 OR 3	319,897
	8	5 OR 6	15,704
	9	4 AND 7 AND 8	**165**
**Scopus**		"social media" OR "social marketing" OR "mass media" AND fruit* OR vegetable* AND "youn g adult*"	**287**
**Embase**	1	‘social marketing’/exp OR ‘social marketing’	3509
	2	‘social media’	4449
	3	‘mass media’	4157
	4	1 or 2 or 3	11,849
	5	‘young adult’ or ‘young adults’	138,234
	6	‘fruits and vegetables’ or ‘fruit’ or ‘vegetable’	162,845
	7	4 and 5 and 6	**6**

**PsycInfo**	1	Adult/ or Young Adult/ or young adult*.mp.	33964
	2	Fruit/ or fruit*.mp.	15808
	3	Vegetable*.mp. or Vegetables/	3748
	4	2 or 3	16715
	5	Social marketing.mp. or social marketing/	1225
	6	Social media. mp or Mass Media/ or Social Media/	11955
	7	5 or 6	13102
	8	1 and 4 and 7	2
	9	Limit 8 to (English language and humans and yr = 1990-current)	**0**
