# Supplementary material for: Efficacy and External Validity of Electronic and Mobile Phone-Based Interventions Promoting Vegetable Intake in Young Adults: Systematic Review and Meta-Analysis
Source: J Med Internet Res. 2016 Apr 8;18(4):e58. doi: 10.2196/jmir.5082 (PMC4841894; doi:10.2196/jmir.5082)
Supplement: Multimedia Appendix 2 [file jmir_v18i4e58_app2.pdf]

Studies excluded by full text with reasons (n=73)

Not using target strategy

1. Campbell MK, Bernhardt JM, Waldmiller M, Jackson B, Potenziani D, Weathers B, et al. Varying the message source in computer-tailored nutrition education. Patient Education and Counseling. 1999 Feb;36(2):157-69. PubMed PMID: WOS:000078789500006.
2. Glanz K, Hersey J, Cates S, Muth M, Creel D, Nicholls J, et al. Effect of a Nutrient Rich Foods consumer education program: results from the nutrition advice study. J Acad Nutr Diet. 2012;112(1):56-63. PubMed PMID: 22717177.
3. Kreausukon P, Gellert P, Lippke S, Schwarzer R. Planning and self-efficacy can increase fruit and vegetable consumption: a randomized controlled trial. Journal of behavioral medicine. 2012;35(4):443-51.
4. Pollard CM, Miller MR, Daly AM, Crouchley KE, O'Donoghue KJ, Lang AJ, et al. Increasing fruit and vegetable consumption: success of the Western Australian Go for 2&5 campaign. Public Health Nutrition. 2008;11(3):314-20. PubMed PMID: 17612423.
5. Sidahmed E, Cornellier ML, Ren J, Askew LM, Li Y, Talaat N, et al. Development of exchange lists for Mediterranean and Healthy Eating Diets: implementation in an intervention trial. Journal of Human Nutrition & Dietetics. 2014;27(5):413-25. PubMed PMID: 2012737297. Language: English. Entry Date: 20141003. Revision Date: 20150116. Publication Type: journal article.
6. Smeets T, Kremers SP, Brug J, de Vries H. Effects of tailored feedback on multiple health behaviors. Ann Behav Med. 2007 Apr;33(2):117-23. PubMed PMID: CN-00636776 UPDATE.

Not RCT

7. Cox RH, White AH, Gaylord CK. A video lesson series is effective in changing the dietary intakes and food-related behaviors of low-income homemakers. J Am Diet Assoc. 2003;103(11):1488-93.
8. Harvey-Berino J, Pope L, Gold BC, Leonard H, Belliveau C. Undergrad and Overweight: An Online Behavioral Weight Management Program for College Students. Journal of Nutrition Education and Behavior. 2012 11//;44(6):604-8.
9. Kothe EJ, Mullan BA, Butow P. Promoting fruit and vegetable consumption. Testing an intervention based on the theory of planned behaviour. Appetite. 2012;58(3):997-1004. PubMed PMID: 22349778.
10. Block G, Block T, Wakimoto P, Block CH. Demonstration of an E-mailed worksite nutrition intervention program. Preventing chronic disease. 2004;1(4):A06.
11. Brug J, Steenhuis I, van Assema P, Glanz K, De Vries H. Computer-tailored nutrition education: differences between two interventions. Health Educ Res. 1999 Apr;14(2):249-56. PubMed PMID: WOS:000079905200010.
12. Corsino L, Lin P-H, Batch BC, Intille S, Grambow SC, Bosworth HB, et al. Recruiting young adults into a weight loss trial: Report of protocol development and recruitment results. Contemporary Clinical Trials. 2013 7//;35(2):1-7.

Review paper

13. Maon S, Edirippulige S, Ware R, Batch J. The use of web-based interventions to prevent excessive weight gain. Journal of Telemedicine and Telecare. 2012;18(1):37-41.

14. Rekhy R, McConchie R. Promoting consumption of fruit and vegetables for better health. Have campaigns delivered on the goals? Appetite. 2014;79:113-23.

Not relevant outcome

15. Ang YK, Mirnalini K, Zalilah MS. A workplace email-linked website intervention for modifying cancer-related dietary and lifestyle risk factors: rationale, design and baseline findings. Malays. 2013;19(1):37-51. PubMed PMID: 24800383.
16. Dour CA, Horacek TM, Schembre SM, Lohse B, Hoerr S, Kattelmann K, et al. Process evaluation of project webhealth: A nondieting web-based intervention for obesity prevention in college students. Journal of Nutrition Education and Behavior. 2013;45(4):288-95.
17. Miskovsky MJ. Lessons Learned When Evaluating Web-based Nutrition Education in College Freshmen. The Journal for Nurse Practitioners. 2012 2//;8(2):123-8.
18. Park A, Nitzke S, Kritsch K, Kattelmann K, White A, Boeckner L, et al. Internet-based interventions have potential to affect short-term mediators and indicators of dietary behavior of young adults. Journal of nutrition education and behavior. 2008 Sep;40(5):288-97. PubMed PMID: CN-00666953 UPDATE.
19. Atkin CK. Effects of televised alcohol messages on teenage drinking patterns. Journal of Adolescent Health Care. 1990 1//;11(1):10-24.
20. Bonfiglioli C, Hattersley L, King L. Australian print news media coverage of sweet, non-alcoholic drinks sends mixed health messages. Australian and New Zealand Journal of Public Health. 2011;35(4):325-30.
21. Chen X, Yang X. Does food environment influence food choices? A geographical analysis through "tweets". Applied Geography. 2014 7//;51(0):82-9.
22. Chiu C-M, Cheng H-L, Huang H-Y, Chen C-F. Exploring individuals' subjective well-being and loyalty towards social network sites from the perspective of network externalities: The Facebook case. International Journal of Information Management. 2013 6//;33(3):539-52.
23. Cruwys T, Bevelander KE, Hermans RCJ. Social modeling of eating: A review of when and why social influence affects food intake and choice. Appetite. 2015;86:3-18.
24. Della LJ, Dejoy DM, Lance CE. Explaining fruit and vegetable intake using a consumer marketing tool. Health education & behavior : the official publication of the Society for Public Health Education. 2009 Oct;36(5):895-914. PubMed PMID: 19158253. Epub 2009/01/23. eng.
25. Epstein SB, Jean-Pierre K, Lynn S, Kant AK. Media coverage and awareness of the 2010 Dietary Guidelines for Americans and MyPlate. American Journal of Health Promotion. 2013;28(1):e30-e9.
26. Graham DJ, Pelletier JE, Neumark-Sztainer D, Lust K, Laska MN. Perceived Social-Ecological Factors Associated with Fruit and Vegetable Purchasing, Preparation, and Consumption among Young Adults. Journal of the Academy of Nutrition and Dietetics. 2013 10//;113(10):1366-74.
27. McKinley CJ, Wright PJ. Informational social support and online health information seeking: Examining the association between factors contributing to healthy eating behavior. Computers in Human Behavior. 2014 8//;37(0):107-16.
28. Simunaniemi AM, Sandberg H, Andersson A, Nydahl M. Laypeople Blog About Fruit and Vegetables for Self-Expression and Dietary Influence. Health communication. 2011;26(7):621-30.
29. Vaterlaus JM, Patten EV, Roche C, Young JA. #Gettinghealthy: The perceived influence of social media on young adult health behaviors. Computers in Human Behavior. 2015 4//;45(0):151-7.
30. Wammes B, Breedveld B, Looman C, Brug J. The impact of a national mass media campaign in The Netherlands on the prevention of weight gain. Public Health Nutrition. 2005;8(8):1250-7.
31. Yahia N, Achkar A, Abdallah A, Rizk S. Eating habits and obesity among Lebanese university students. Nutrition Journal. 2008;7(1).
32. Melton BF, Bigham LE, Bland HW, Bird M, Fairman C. Health-related behaviors and technology usage among college students. Am J Health Behav. 2014;38(4):510-8.

33. Lohse B. Facebook Is an Effective Strategy to Recruit Low-income Women to Online Nutrition Education. Journal of Nutrition Education and Behavior. 2013;45(1):69-76.

Not relevant population

34. Hollis JF, Gullion CM, Stevens VJ, Brantley PJ, Appel LJ, Ard JD, et al. Weight Loss During the Intensive Intervention Phase of the Weight-Loss Maintenance Trial. Am J Prev Med. 2008 8//;35(2):118-26.
35. Petersen R, Sill S, Lu C, Young J, Edington DW. Effectiveness of employee internet-based weight management program. Journal of Occupational & Environmental Medicine. 2008;50(2):163-71. PubMed PMID: 18301173.
36. Alexander GL, McClure JB, Calvi JH, Divine GW, Stopponi MA, Rolnick SJ, et al. A randomized clinical trial evaluating online interventions to improve fruit and vegetable consumption. Am J Public Health. 2010;100(2):319-26. PubMed PMID: 2010534521. Corporate Author: MENU Choices Team. Language: English. Entry Date: 20100226. Revision Date: 20101231. Publication Type: journal article.
37. Anderson ES, Winett RA, Wojcik JR, Winett SG, Bowden T. A computerized social cognitive intervention for nutrition behavior: Direct and mediated effects on fat, fiber, fruits, and vegetables, self-efficacy, and outcome expectations among food shoppers. Ann Behav Med. 2001 Spr;23(2):88-100. PubMed PMID: WOS:000168707600003.
38. Block G, Sternfeld B, Block CH, Block TJ, Norris J, Hopkins D, et al. Development of Alive! (A Lifestyle Intervention Via Email), and its effect on health-related quality of life, presenteeism, and other behavioral outcomes: randomized controlled trial. J Med Internet Res. 2008;10(4):e43. PubMed PMID: 19019818. Pubmed Central PMCID: PMC2629370.
39. Buller DB, Woodall WG, Zimmerman DE, Slater MD, Heimendinger J, Waters E, et al. Randomized trial on the 5 a day, the Rio Grande Way Website, a web-based program to improve fruit and vegetable consumption in rural communities. J Health Commun. 2008 Apr;13(3):230-49. PubMed PMID: CN-00640380 UPDATE.
40. Buller MK, Kane IL, Dunn AL, Edwards EJ, Buller DB, Liu X. Marketing fruit and vegetable intake with interactive games on the Internet. Social Marketing Quarterly. 2009 2009;15(Suppl. 1):136-54. PubMed PMID: CABI:20093219677.
41. Campbell MK, Resnicow K, Carr C, Wang T, Williams A. Process evaluation of an effective church-based diet intervention: Body & soul. Health Education and Behavior. 2007;34(6):864-80.
42. Carlson JA, Sallis JF, Ramirez ER, Patrick K, Norman GJ. Physical activity and dietary behavior change in Internet-based weight loss interventions: comparing two multiple-behavior change indices. Prev Med. 2012;54(1):50-4. PubMed PMID: 22085706. Pubmed Central PMCID: NIHMS336290 PMC3254700.
43. Carpenter KM, Lovejoy JC, Lange JM, Hapgood JE, Zbikowski SM. Outcomes and utilization of a low intensity workplace weight loss program. J Obes. 2014;2014:414987. PubMed PMID: 24688791. Pubmed Central PMCID: PMC3941961. Epub 2014/04/02. eng.

44. Colby SE, Olfert M, Mathews D, Kattelmann KK, Franzen-Castle L, White A. iCook: Development of web-based component of a 4-H cooking intervention. Faseb Journal. 2013 Apr;27. PubMed PMID: WOS:000319860502448.
45. Hutchesson MJ, Collins CE, Morgan PJ, Watson JF, Guest M, Callister R. Changes to dietary intake during a 12-week commercial web-based weight loss program: A randomized controlled trial. Eur J Clin Nutr. 2014;68(1):64-70.
46. Jacobs N, Clays E, De Bacquer D, De Backer G, Dendale P, Thijs H, et al. Effect of a tailored behavior change program on a composite lifestyle change score: a randomized controlled trial. Health Educ Res. 2011 Oct;26(5):886-95. PubMed PMID: CN-00811040 UPDATE.

47. Johnson SS, Paiva AL, Cummins CO, Johnson JL, Dyment SJ, Wright JA, et al. Transtheoretical model-based multiple behavior intervention for weight management: effectiveness on a population basis. Prev Med. 2008 Mar;46(3):238-46. PubMed PMID: CN-00637269 UPDATE.
48. Kim Y, Pike J, Adams H, Cross D, Doyle C, Foreyt J. Telephone intervention promoting weight-related health behaviors. Prev Med. 2010 3//;50(3):112-7.
49. Lange D, Richert J, Koring M, Knoll N, Schwarzer R, Lippke S. Self-regulation prompts can increase fruit consumption: a one-hour randomised controlled online trial. Psychol Health. 2013;28(5):533-45. PubMed PMID: CN-00906389 UPDATE.
50. Luszczynska A, Tryburcy M, Schwarzer R. Improving fruit and vegetable consumption: a self-efficacy intervention compared with a combined self-efficacy and planning intervention. Health Educ Res. 2007 Oct;22(5):630-8. PubMed PMID: 17060349. Epub 2006/10/25. eng.
51. Mouttapa M, Robertson TP, McEligot AJ, Weiss JW, Hoolihan L, Ora A, et al. The Personal Nutrition Planner: A 5-Week, Computer-tailored Intervention for Women. Journal of Nutrition Education and Behavior. 2011 May-Jun;43(3):165-72. PubMed PMID: WOS:000291203300009.
52. Neuenschwander LM, Abbott A, Mobley AR. Comparison of a web-based vs in-person nutrition education program for low-income adults. Journal of the Academy of Nutrition and Dietetics. 2013 Jan;113(1):120-6. PubMed PMID: CN-00912294 UPDATE.
53. Nolan RP, Upshur RE, Lynn H, Crichton T, Rukholm E, Stewart DE, et al. Therapeutic benefit of preventive telehealth counseling in the Community Outreach Heart Health and Risk Reduction Trial. American journal of cardiology. 2011 Mar;107(5):690-6. PubMed PMID: CN-00780249 UPDATE.
54. Norman GJ, Kolodziejczyk JK, Adams MA, Patrick K, Marshall SJ. Fruit and vegetable intake and eating behaviors mediate the effect of a randomized text-message based weight loss program. Preventive Medicine. 2013;56(1):3-7. PubMed PMID: 2011896669. Language: English. Entry Date: 20130628. Revision Date: 20130628. Publication Type: journal article.
55. O'Brien KM, Hutchesson MJ, Jensen M, Morgan P, Callister R, Collins CE. Participants in an online weight loss program can improve diet quality during weight loss: A randomized controlled trial. Nutrition Journal. 2014;13(1).
56. Oenema A, Brug J. Feedback strategies to raise awareness of personal dietary intake: results of a randomized controlled trial. Prev Med. 2003 4//;36(4):429-39.
57. Oenema A, Brug J, Lechner L. Web-based tailored nutrition education: results of a randomized controlled trial. Health Educ Res. 2001 Dec;16(6):647-60. PubMed PMID: CN-00376828 UPDATE.
58. Oenema A, Tan F, Brug J. Short-term efficacy of a web-based computer-tailored nutrition intervention: main effects and mediators. Ann Behav Med. 2005 Feb;29(1):54-63. PubMed PMID: CN-00513840 UPDATE.
59. Papadaki A, Scott JA. The Mediterranean eating in Scotland experience project: evaluation of an Internet-based intervention promoting the Mediterranean diet. Br J Nutr. 2005;94(2):290-8. PubMed PMID: 16115365.
60. Parekh S, King D, Boyle FM, Vandelanotte C. Randomized controlled trial of a computer-tailored multiple health behaviour intervention in general practice: 12-month follow-up results. International Journal of Behavioral Nutrition and Physical Activity. 2014;11(1).
61. Patrick K, Calfas KJ, Norman GJ, Rosenberg D, Zabinski MF, Sallis JF, et al. Outcomes of a 12-month web-based intervention for overweight and obese men. Ann Behav Med. 2011 Dec;42(3):391-401. PubMed PMID: CN-00920278 UPDATE.
62. Pratt DS, Jandzio M, Tomlinson D, Kang X, Smith E. The 5-10-25 challenge: an observational study of a web-based wellness intervention for a global workforce. Disease Management. 2006;9(5):284-90. PubMed PMID: 17044762.
63. Risica PM, Strolla LO, Fournier L, Kirtania U, Upegui D, Zhao J, et al. Effectiveness of different methods for delivering tailored nutrition education to low income, ethnically diverse adults. International Journal of Behavioral Nutrition and Physical Activity. 2009;6.
64. Rossi MC, Perozzi C, Consorti C, Almonti T, Foglini P, Giostra N, et al. An interactive diary for diet management (DAI): a new telemedicine system able to promote body weight reduction, nutritional

education, and consumption of fresh local produce. Diabetes Technology & Therapeutics. 2010;12(8):641-7. PubMed PMID: 20615106.
65. Schulz DN, Kremers SPJ, van Osch LADM, Schneider F, van Adrichem MJG, de Vries H. Testing a Dutch web-based tailored lifestyle programme among adults: a study protocol. BMC Public Health. 2011 Feb 16;11. PubMed PMID: WOS:000288071800001.
66. Shaikh AR, Vinokur AD, Yaroch AL, Williams GC, Resnicow K. Direct and mediated effects of two theoretically based interventions to increase consumption of fruits and vegetables in the healthy body healthy spirit trial. Health Education and Behavior. 2011;38(5):492-501.
67. Springvloet L, Lechner L, De Vries H, Candel MJJM, Oenema A. Short-and medium-term efficacy of a web-based computer-tailored nutrition education intervention for adultsincluding cognitive and environmental feedback: Randomized controlled trial. Journal of Medical Internet Research. 2015;17(1):e23.
68. Sternfeld B, Block C, Quesenberry Jr CP, Block TJ, Husson G, Norris JC, et al. Improving Diet and Physical Activity with ALIVE: A Worksite Randomized Trial. Am J Prev Med. 2009 6//;36(6):475-83.
69. Thomas D, Vydelingum V, Lawrence J. E-mail contact as an effective strategy in the maintenance of weight loss in adults. Journal of human nutrition and dietetics. 2011 Feb;24(1):32-8. PubMed PMID: CN-00771049 UPDATE.
70. van Keulen HM, Mesters I, Ausems M, van Breukelen G, Campbell M, Resnicow K, et al. Tailored print communication and telephone motivational interviewing are equally successful in improving multiple lifestyle behaviors in a randomized controlled trial. Annals of behavioral medicine : a publication of the Society of Behavioral Medicine. 2011;41(1):104-18.
71. Wharton CM, Johnston CS, Cunningham BK, Sterner D. Dietary Self-Monitoring, But Not Dietary Quality, Improves With Use of Smartphone App Technology in an 8-Week Weight Loss Trial. Journal of Nutrition Education and Behavior. 2014 9//;46(5):440-4.
72. Woodall WG, Buller DB, Saba L, Zimmerman D, Waters E, Hines JM, et al. Effect of emailed messages on return use of a nutrition education website and subsequent changes in dietary behavior. J Med Internet Res. 2007;9(3):e27. PubMed PMID: CN-00705372 UPDATE.

Abstract only

73. Hivert MF, Doyon M, Brown C, Cuerrier JP, Carpentier A, Langlois MF. An educational intervention combining small-group seminars and emails prevents weight gain in young adults over the first two years of university. Obesity. 2010;18:S84-S5.
