# Supplementary material for: Efficacy and External Validity of Electronic and Mobile Phone-Based Interventions Promoting Vegetable Intake in Young Adults: Systematic Review and Meta-Analysis
Source: J Med Internet Res. 2016 Apr 8;18(4):e58. doi: 10.2196/jmir.5082 (PMC4841894; doi:10.2196/jmir.5082)
Supplement: Multimedia Appendix 3 [file jmir_v18i4e58_app3.pdf]

Table S3: Study descriptions of reach and representativeness of participants (n=14)

Authors, year, country, citation	Target audience	Recruitment methods	Participation rate (%)	Key inclusion criteria	*N* (study arms)	Baseline characteristics
Clifford *et al.* (2009) USA (69)	College students living off campus	Students volunteered from non-nutrition courses	60.8	NR	101 (I=50, C=51)	Age: NR; Gender: M, F=63%; 93% live off campus; 52% cook dinner ≥4 times/week
Franko *et al.* (2008) USA (73)	College students aged 18-24 yrs	Information and recruitment table on University grounds	79.0	Age: 18-24 yo; enrolled as full-time undergraduate student; not dieting	476 (group I=165, group II= 164, C=147)	Age: I=20.1 +/-1.7 yrs. II=20.0 +/ 1.7 yrs. C=20.1 +/-1.7 yrs; 58.2% White; Gender: M,F=52.3%
Gow *et al.*(2010) USA (67)	First year college students aged 22 yrs or younger	Recruited from Psychology courses through classroom announcements &fliers	94.6	Age: ≤22 yrs; First year college students.	170 (internet=40, feedback=39, combined=40, control=40)	Age: 18.1 yrs; Gender: M, F=74%; 53.8% White; 60.8% Living in dormitory ; Mean BMI: 24.38kg/m^2
Greene *et al.* (2012) USA (62)	College students aged 18-24 yrs	Flyers, table tents in dining halls, newspaper ads, online and class announcements.	64.4	Age: 18 to 24 yo; BMI: >18.5 kg/m^2; healthy; not pregnant, lactating or studying nutrition or exercise science	1689 (I=830, C=859)	Age: Completers 19.07 yrs +/- 1.1, Non-completers 19.2 yrs +/- 1.2; Gender: M, F=62%; 79% White
Hebden *et al.* (2013) Australia (65)	Young adults aged 18-35 yrs from university population	Advertisements posted around the university and published in staff and student newsletters	92.7	Age: 18–35 yo; BMI 24–31.99 or 23–23.99 kg/m^2 with weight gain >2 kg in past 12 months; can receive SMS; have regular internet access; not dieting, pregnant or planning pregnancy in next 3 months; no medical condition that influences body weight.	51 (I=26, C=25)	Age: C= 23.1 +/-3.7 yrs, I=22.6 +/-5.4 yrs; Gender: M, F=80.4%; Lives with parents/other: 53%
Kattelmann *et al.* (2014) USA (63)	College students aged 18-24 yrs	In-class and campus housing meetings, e-mails, letters, and flyers on campuses	49.2	Age: 18–24 yo; full-time 1st-3rd yr student with access to internet; not studying nutrition, exercise, or health promotion. BMI >18.5 kg/m^2; healthy; not pregnant.	1,639 (I=824 C= 815)	Age: 19.3 +/- 1.1 yrs; 72.1 % White; 73.8% live on campus; 11.5% consuming >5 cups fruit and veg/day
Kothe and Mullan (2014) Australia (68)	First year undergrad psychology students	Recruited as part of psychology course	NR	NR	162 (I=81, C=81)	Mean Age: 18.84 yrs; Gender: M, F=83.3%; 78.4% Living with parents; 46.9% Australian, 25.3% North Asian

Authors, year, country, citation	Target audience	Recruitment methods	Participation rate (%)	Key inclusion criteria	*N* (study arms)	Baseline characteristics
Kypri and McAnally (2005) New Zealand (74)	17-24 yos attending university health service	Patients attending university student health service invited to participate	82.0	NR	218 (group A=72, group B=74, group C=72)	Mean Age: 20.2 +/- 1.5 yrs; Gender: M, F=49%; 75% European, 8% Maori
LaChausse (2012) USA (71)	Undergradu-ate University students	E-mail messages, flyers and announcements on school billboards.	89.4	NR	312 (MSB=106, Campus=70, C=136)	Age: MSB=26.7 +/- 9.8 yrs, Campus=25.1+/-8.9 yrs, C=22.8+/-6.4 yrs; Gender: M,F=75.6% 44% Hispanic, 21.2% White
Nitzke *et al.* (2007) USA (64)	Young adults aged 18-24 yrs	Personal contacts and standardized posted ads with toll-free phone numbers	100	Age: 18–24 yo; not enrolled in a nutrition program in previous 12 months, limited income (receiving welfare or income <16,000 PA, if college student-paying own college expenses)	2024 (I=1004, C= 1020)	Mean Age= 20.6 yrs; Gender: M,F=61.2%; 53.7% Caucasian, 27.1% African American; 41% live with parents; 86% responsible for food preparation
Partridge *et al.* (2015) Australia (66)	Young adults aged 18-35 yrs	Invitations from participating doctors, electronic or print ads, university newsletters, posters, mailbox drops and newspapers	64.4	18-35 yo, BMI 25-31.9 kg/m^2, or 23-24.9 kg/m^2 with weight gain >2 kg in last 12 months; fruit intake >2 servings daily; vegetable intake >5 servings daily; SSB intake ≥1 L weekly; takeout food> once/week; and/or engaged in moderate-intensity PA <60 minutes daily.	250 (I=125, C=125)	Mean Age: 27.7 yrs; Gender: M, F=61.7%; 69.4% English speaking only; 75.4% living in socially advantaged area
Richards *et al.* (2006) USA (75)	College students aged 18-24 yrs	NR	NR	Age: 18-24 yo; non-dietetic major; have current e-mail, mail address, and telephone number.	314 (I=157, C=157)	Age: 20.4 +/-1.5 yrs; Gender: M, F=75.2%; 96.8% White
Rompotis *et al.* (2014) Australia (72)	Undergradu-ate psychology students	Electronic bulletin board	NR	Age: 18-34 yo; own a mobile phone and a student email account	161 (email I=30, email C1=29, email C2=29, SMS I=26, SMS C1=24, SMS C2=23)	Mean age=19.5 yrs; Gender: M, F= 81.7%
Shahril *et al.* (2013) Malaysia (70)	18 and 24 yo University students.	Students recruited from class lists based on study eligibility criteria	NR	Age: 18-24 yo; actively using mobile phone, first or second year diploma or degree from management studies; healthy and able to read, write, speak, and understand Malay or English	417 (I = 205, C= 212)	Mean Age=19.1 yrs; Gender: M, F= 87.6%

BMI, body mass index; C, control; C1, control group 1; C2, control group 2; F, female; I, intervention; M, male; MSB, my student body; NR, not reported; PA, physical activity; SMS, short message service; SSB, sugar sweetened beverage; yrs, years, yo, years old
