# Supplementary material for: Efficacy and External Validity of Electronic and Mobile Phone-Based Interventions Promoting Vegetable Intake in Young Adults: Systematic Review and Meta-Analysis
Source: J Med Internet Res. 2016 Apr 8;18(4):e58. doi: 10.2196/jmir.5082 (PMC4841894; doi:10.2196/jmir.5082)
Supplement: Multimedia Appendix 4 [file jmir_v18i4e58_app4.pdf]

Table S4: Study Description of intervention implementation and adaption (n=14)

Author, year, citation	Intervention type	Intervention setting	Intervention description<br>Comparator description	Number of sessions<br>Delivery expertise	Intervention/ follow-up post intervention	Adherence to protocol
Clifford *et al*. 2009 (68)	Nutrition-oriented cooking show based on SCT	Individual<br>University (n=1)<br>Online	I: view 4 15-minute cooking programs<br>C: view 4 5-minute programs on sleep disorders.	I: 4x15-minute videos<br>C: 4x5-minute videos<br>Dietician (n=1)	4 wks<br>4 months	NR
Franko *et al*. (2008) (72)	Theory/ education based with goal setting	Individual<br>University (n=6)<br>Online	I: Exposure to web nutrition education (text based audio info, interactive activities and goal setting)<br>C: Exposure to interactive anatomy education website	I: experimental I - 2 online sessions, experimental II- 2 online sessions + 1 booster C:2 control web sessions.<br>RS (n=NR)	2 wks<br>6 months	I: 94% completed sessions and post test<br>II: 93.3% completed sessions and post test
Gow *et al*.(2010) (66)	Theory/ education based with monitoring and feedback based on SOC (TTM)	Individual<br>University (n = 1)<br>Online<br>Email	I: Online intervention on healthy eating and exercise<br>FI: weight and caloric feedback only (via email)<br>CI: Combined feedback and online intervention<br>C: no treatment	I: 6× approx. 45 min weekly<br>FI: 6× weekly emails in response to self-reported weight<br>CI: both 1 and 2<br>NR	6 wks<br>3 months	Significant differences between groups attending ≥4 sessions (CI 82.1%; I 65.8% and FI 89.9%) >3 session required
Greene *et al*. (2012) (61)	Theory/ education based with goal setting and feedback	Individual<br>University (n = 8)<br>Online	I: Individualized online profile page with feedback on current intake vs recommended. Access to web-based nutrition and PA curriculum based on non-diet principles<br>C: Profile page only.	10 × 15 min weekly online lessons<br>RS (n=NR)	12 wks<br>15 months	84% of intervention group completed 10/10 sessions. 5.1% didn't complete any sessions
Hebden *et al*. (2013) (64)	Behavior change based SOC (TTM). Monitoring and feedback with goal setting	Individual<br>Mobile phone<br>Online<br>Email	I: Diet booklet with instructions. Participants selected 2/4 lifestyle behaviors e.g. F&V intake. Received SMS, emails based on SOC, and access to phone apps and web forums.<br>C: Diet booklet only.	1 initial in-person consultation; 48 SMS (4/wk); 48 emails (4/wk)<br>Unlimited access to apps and Internet Forums<br>Dietician (n=1)	12 wks<br>No f/u	99.6% SMS delivery,<br>48.8% replies with 13/26 replying to over half,<br>100% email delivery
Kattelmann *et al*. (2014) (62)	Theory/ education based using SOC (TTM) tailoring and goal setting	Individual<br>University (n = 13)<br>Online<br>Email	I: Access to voluntary mini educational lessons & e-mails with personalized videos tailored to SOC, reinforcing lesson and encouraging to set goals.<br>C: Access to material after f/u assessment	I: 21 educational lessons<br>4 nudges per wk (1 as a reminder to view lesson)<br>Dieticians (n=NR)	10 wks<br>12 months	I: 75% completed 10 wk intervention.
Kothe and Mullan (2014) (67)	Promoting behavior incorporating theory of planned behavior	Individual<br>University (n = 1)<br>Email	I: Received emails promoting F&V consumption. Messages targeted attitude, subjective norm, and perceived behavioral control.<br>C: no exposure to emails	I: Emails every 3 days over 1 month<br>RS (n=NR)	4 wks<br>No f/u	NR

Author, year, citation	Intervention type	Intervention setting	Intervention description Comparator description	Number of sessions Delivery expertise	Intervention/ follow-up post intervention	Adherence to protocol
Kypri and McAnally (2005) (73)	Feedback based on authority recommendat-ions and social norms	Individual University (n = 1) Online, Email	I: web-based assessment and personalized feedback using health authority recommendations, social norms and self-comparison<br>C1: assessment only<br>C2: minimal contact	1 session<br>RS (n=NR)	6 wks<br>No f/u	NR
LaChausse (2012) (70)	Theory/ education based Individualized feedback	Individual University (n = 8) Online	I: Online nutrition education course<br>C1: on-campus nutrition education course, reflection papers on course discussions and exams.<br>C2: no exposure	I: 2 hours/ wk over 12-weeks<br>C1: 1 2hr session/wk over 12 wks<br>Health promotion officer (n=1)	12 wks<br>2 wks	I: logged into the online course M= 29.84 times (SD = 12.44) over 12wks.<br>C1: NR
Nitzke *et al*. (2007) (63)	Individualized feedback based on SOC (TTM)	Individual home based 10 states Phone calls	I: mailed materials (individualized feedback on current intake & advice based on SOC) and phone calls enforcing material<br>C: mailed non-tailored pamphlet.	I: 2 phone calls over 6 months and series of mailed material<br>RS & outreach educators (n=NR)	6 months<br>6 months	NR
Partridge *et al*. (2015) (65)	Behavior Change based on SOC (TTM) Monitoring and feedback with goal setting	Individual Mobile phone Online Email SMS	I: Received 18 page diet booklet and 8 SMS, 1 email weekly based on SOC, and 5 personalized coaching calls, access to phone apps, blog, website<br>C: Received printed dietary and physical activity guidelines and 4 SMS	I: 8 SMS, 1 email weekly, 5 personalized coaching calls (10-15 mins each, 25 mins for initial)<br>C: 1 SMS every 3 wks during wk 1-12<br>Dietician (n=2)	12 wks<br>6 months (only 12 wk data available)	4.6/5 coaching calls completed, 53.7% replied to SMS requiring response, 76.4% use of emails, 74.5% didn't use the apps, 65.5% used diet booklet, 59.1% didn't use website
Richards *et al*. (2006) (74)	Individually tailored motivational interviewing based on SOC (TTM), with goal setting	Individual University (n=1) Online Email Phone calls	I: received stage-based newsletters, motivational interview (by phone) to identify barriers and solutions to F&V consumption, tailored e-mails: recipes, nutrition facts, F&V tips, Websites<br>C: assessment only	I: 4 newsletters, 1 motivational interview, minimum of 2 emails<br>Dietician (n=1)	4 months<br>No f/u	52.3% visited website,<br>95.5% received motivational interview phone call
Rompotis *et al*. (2014) (71)	Habit formation	Individual University (n=1) Mobile phone Email	I: F&V intake-habit formation messages 1) via emails 2) via SMS<br>C1: general F&V messages 1) via emails 2) via SMS<br>C2:general healthy eating messages 1) via emails 2) via SMS	1: 24 emails<br>2: 24 SMS<br>Over 8 wks<br>RS (n=NR)	8 wks<br>8 wks	NR
Shahril *et al*. (2013) (69)	Theory/ Education Based	Individual University (n=4) SMS	I: Lecture on dietary guidelines, brochures and SMS enforcing information<br>C: No exposure	I: 1 SMS every 5 days + 1 hour nutrition lecture + 3 pamphlets<br>RS, nutritionist (n=1)	10 wks<br>No f/u	NR

C, control; C1, control group 1; C2, control group 2; CI, combined intervention; FI, feedback intervention; f/u, follow up; F&V, fruit and vegetables; I, intervention; II, intervention group 2; M, mean; NR, not reported; PA, physical activity RS, research staff; SCT, social cognitive theory; SMS, short message service; SOC, stage of change; TTM, transtheoretical model; wks, weeks
