# Supplementary material for: Efficacy and External Validity of Electronic and Mobile Phone-Based Interventions Promoting Vegetable Intake in Young Adults: Systematic Review and Meta-Analysis
Source: J Med Internet Res. 2016 Apr 8;18(4):e58. doi: 10.2196/jmir.5082 (PMC4841894; doi:10.2196/jmir.5082)
Supplement: Multimedia Appendix 5 [file jmir_v18i4e58_app5.pdf]

Table S5: Study maintenance and institutionalization (n=14)

Author, year, citation	Attrition	Control vs. Intervention (% differential attrition)	Compared drop outs	Long term effects	Program sustainability
Clifford *et al.* 2009 (69)	NR	NR	No differences between completers and non-completers.	Changes not maintained at 4 month follow up	NR
Franko *et al.* (2008) (73)	26.7	30.6 vs 23.6 (I), 26.1 (II)	NR	Changes not maintained at 6 month follow up	NR
Gow *et al.*(2010) (67)	20.8	20 vs. 43.9 (II);<br>41.0 (FI) and 20.5 (CI)	Drop outs reported less F&V consumption than completers ($P < 0.5$)	Changes not maintained at 3 month follow up	NR
Greene *et al.* (2012)(62)	20.2 (12 wks)<br>33.3 (15 mo)	17.7 vs. 22.9 (12 wks)<br>31.2 vs 35.5 (15 mo)	A greater proportion of completers were white ($P< 0.05$) and had a lower BMI at baseline ($P< 0.05$)	Changes not maintained at 15 month follow up	NR
Hebden *et al.* (2013) (65)	9.8	0 vs 19.3	NR	No follow up	Results used to refine mhealth for larger study in a broader young adult population.
Kattelmann *et al.* (2014) (63)	24.3	23.6 vs 25	More completers (70.4% vs 60.7%) were female and had never used cigarettes (71.7% vs 65.6%).	Changes not maintained at 12 month follow up	NR
Kothe and Mullan (2014) (68)	18.5	17.3 vs. 19.8	No differences between completers and non-completers.	No follow up	NR
Kypri and McAnally (2005) (74)	13.3	12.5 vs. 15.3 (I), 12.2 (II)	Greater proportion of smokers among non-completers ($P< 0.05$).	No follow up	NR
LaChausse (2012) (71)	2.5	NR	NR	NR	NR
Nitzke *et al.* (2007) (64)	38 (12 months)	NR	Education less than high school completion, non-White ethnicity, male gender, living with children, and income ≤ $800/month predicted attrition ($P< .001$).	Changes maintained at 6 month follow up 12 month post-intervention NR.	Sustainability of changes measured at 6 months but not beyond, and not for broader young adult population.
Partridge *et al.* (2015) (66)	8.0	0 vs 8.0	No demographic differences between completers and non-completers. However non-completers consumed more take away food at baseline.	Study still underway	Study still underway
Richards *et al.* (2006)(75)	28	NR	More female completers ($P<.001$)	No follow up	NR
Rompotis *et al.* (2014) (72)	55.3	52.0 (C1)email, 62.0 (C2)email vs. 47.0 (I)email;<br>62.0 (C1)SMS, 57.0 (C2)SMS vs. 54.0 (I)SMS	No significant differences found between completers and non-completers.	NR	NR
Shahril *et al.* (2013) (70)	8.9	4.7 vs. 13.2	NR	No follow up	NR

C, control; C1, control group 1; C2, control group 2; CI, combined intervention; FI, feedback intervention; F&V, fruit and vegetables; I, intervention; II, intervention group 2; Mo, months; NR, not reported; vs, versus; wks, weeks
