# Supplementary material for: Efficacy and External Validity of Electronic and Mobile Phone-Based Interventions Promoting Vegetable Intake in Young Adults: Systematic Review and Meta-Analysis
Source: J Med Internet Res. 2016 Apr 8;18(4):e58. doi: 10.2196/jmir.5082 (PMC4841894; doi:10.2196/jmir.5082)
Supplement: Multimedia Appendix 6 [file jmir_v18i4e58_app6.pdf]

Table S6. Risk of bias as assessed by the Cochrane Collaboration Tool for included studies (n=14)

First author, Citation	Selection bias									
	Random sequence generation		Allocation concealment		Attrition bias		Detection bias		Reporting bias	
	Cochrane judgment	Supporting evidence	Cochrane judgment	Supporting evidence	Cochrane judgment	Supporting evidence	Cochrane judgment	Supporting evidence	Cochrane judgment	Supporting evidence
Clifford et al. (2009)	Unclear risk	Method of randomization not reported	Unclear risk	Method not described	Unclear risk	Unclear if intention to treat analysis performed	Unclear risk	Insufficient information to determine if researchers or participants were blinded to allocation of participants	Low risk	All pre-specified outcomes were reported
Franko et al (2008)	Low risk	Software program used. No further details specified.	Unclear risk	Method not described	High risk	Intention to treat analysis not performed; Missing data not dealt with appropriately (direct likelihood estimation technique used however this is not data missing at random)	High risk	Research assistants aware of allocation	Low risk	All pre-specified outcomes were reported
Gow et al. (2010)	Low risk	Software program used. No further details specified.	Unclear risk	Method not described	Low risk	Intention to treat analysis performed by assigning dropouts (18/40 in the Internet group, 16/39 in the feedback group, 8/40 in the combined group, 8/40 in the control) their baseline results	Unclear risk	Insufficient information to determine if researchers or participants were blinded to allocation of participants	Low risk	All pre-specified outcomes were reported
Greene et al. (2012)	Unclear risk	Method of randomization not reported, however stratified by institution and gender	Unclear risk	Method not described	High risk	Intention to treat analysis not performed (18 control subjects exposed to intervention and excluded from outcome analysis)	Unclear risk	Insufficient information to determine if researchers or participants were blinded to allocation of participants	Low risk	All pre-specified outcomes were reported
Hebden et al. (2013)	Low risk	Computer software used to generate random sequence	Unclear risk	One investigator supervised randomization but concealment not described	Low risk	Intention to treat analysis performed by imputing baseline values for missing follow-up data (5/26 dropouts in intervention group; 3 disontinued;2 unable to attend follow-up; 0 lost in control group)	High risk	Assessors were not blinded to allocation	Low risk	All pre-specified outcomes were reported
Kattelmann et al (2014)	Low risk	Randomized via a computer-generated program.	Unclear risk	Method not described	Unclear risk	Completers and non-completers compared statistically however not specified if non-completers included in analysis	Unclear risk	Insufficient information to determine if researchers or participants were blinded to allocation of participants	Low risk	All pre-specified outcomes were reported
Kothe and Mullan (2014)	Low risk	Participants were computer randomized to the intervention or control group.	Unclear risk	Method not described	Unclear risk	Unclear if intention to treat analysis performed	Unclear risk	Insufficient information to determine if researchers or participants were blinded to allocation of participants	Low risk	All pre-specified outcomes were reported
Kypri and McAnally (2005)	Low risk	Participants were assigned by a computerized random number generator in blocks of 15 (five per trial arm).	Low risk	Allocation concealment achieved by not informing participants that they were participating in an intervention, and research assistant recruiting was not informed of allocation- done by computer.	High risk	Intention to treat analysis not performed. Missing data for group C at baseline not adjusted.	Low risk	Researchers and participants were blinded to allocation	Low risk	All pre-specified outcomes were reported
LaChausse (2012)	Unclear risk	Method of randomization not reported	High risk	Participants made aware of randomized control study design in orientation session	High risk	Intention to treat analysis not performed, 8 Non-completers of post-test survey excluded from analyses	High risk	Orientation explained the 3 arms of the study to all participants thus blinding was not possible	Low risk	All pre-specified outcomes were reported
Nitzke et al. (2007)	Unclear risk	Method of randomization not reported	Unclear risk	Method not described	Low risk	Intention to treat analysis performed by using baseline data for non-completers at 12 months	Unclear risk	Insufficient information to determine if assessors or participants were blinded to allocation (assessors were from independent) survey	Low risk	All pre-specified outcomes were reported

First author, Citation	Selection bias				Attrition bias		Detection bias		Reporting bias	
	Random sequence generation		Allocation concealment							
	Cochrane judgment	Supporting evidence	Cochrane judgment	Supporting evidence	Cochrane judgment	Supporting evidence	Cochrane judgment	Supporting evidence	Cochrane judgment	Supporting evidence
								center however)		
Partridge et al. (2015)	Low risk	Computer software used to generate random sequence by independent researcher	Low risk	Randomization by independent researcher, allocation concealed from investigators Participants aware of 2 groups but nature of control arm concealed to prevent detection of allocation	Low risk	Intention to treat analysis performed on missing data	Low risk	Researchers and participants were blinded to allocation	Low risk	All pre-specified outcomes were reported
Richards et al. (2006)	Unclear risk	Method of randomization not reported	Unclear risk	Method not described	High risk	Intention to treat analysis not performed. Non-completers excluded from analyses	Unclear risk	Insufficient information to determine if assessors or participants were blinded to allocation	Low risk	All pre-specified outcomes were reported
Rompotis et al. (2014)	Low risk	Randomized using a random number generator through Research Randomizer	Unclear risk	Method not described	High risk	Only the 71 completers were included in analyses with no intention to treat analyses performed	Unclear risk	Insufficient information to determine if assessors or participants were blinded to allocation	Low risk	All pre-specified outcomes were reported
Shahril et al. (2013)	Low risk	Randomized by drawing sealed envelopes containing group assignment.	Unclear	Investigators could not foresee assignment because sealed envelopes containing group assignment were used. However unclear if participants aware of intervention arms	High risk	Intention to treat analysis not performed, dropout was not balanced between groups (27/205 in intervention group and 10/212 in control group)	Low risk	Assessor who was dealing with data was blinded to allocation	Low risk	All pre-specified outcomes were reported
