# Supplementary material for: Efficacy and External Validity of Electronic and Mobile Phone-Based Interventions Promoting Vegetable Intake in Young Adults: Systematic Review and Meta-Analysis
Source: J Med Internet Res. 2016 Apr 8;18(4):e58. doi: 10.2196/jmir.5082 (PMC4841894; doi:10.2196/jmir.5082)
Supplement: Multimedia Appendix 7 [file jmir_v18i4e58_app7.pdf]

Table S7: Change in fruit and vegetable intake between baseline and follow-up for intervention and control arms with calculated effect size Cohen's d (95% CI) (n=14)

**Author (year), citation**	**e/mHealth strategies**	**Results**	
		**Baseline to post intervention**	**Effect Size Cohen's d (95% CI)**
Clifford *et al*. (2009) (69)	Online cooking videos	*Data as mean (SE) F&V serves/day*<br>**Intervention:** pre: 2.82 (0.25), post: 2.46 (0.30)<br>**Control:** pre: 2.67 (0.25), post: 2.77 (0.29)<br>***P*** = NS (*p* >0.05)	*F&V*<br>-0.15 (-0.54; 0.24)
Franko *et al*. (2008) (73)	Online education	*Data as mean (SE) F&V serves/day*[1]<br>**Intervention** I: pre: 3.2 (0.1), post: 3.65, II: pre: 3.0, post: 3.66<br>**Control:** pre: 2.9 (0.1), post: 3.07<br>***P*** <0.01	N/A<br>*(No SE reported post intervention for calculation)*
Gow *et al*. (2010) (67)	Online education, Email	*Data as mean (pre: SD, post: SE) F&V score (value of score not defined)*<br>**Intervention + FB group:** pre: 1.32 (1.67), post: 1.73 (0.21),<br>**Intervention only:** pre: 1.87 (1.54), post: 1.63 (0.21),<br>**FB only:** pre: 1.46 (1.64), post:1.57 (0.22)<br>**Control:** pre: 1.80 (1.43), post: 1.44 (0.21)<br>***P*** = NS (*p* value NR)	*F&V*<br>I + FB: 0.22 (-0.22; 0.66)<br>I only: 0.14 (-0.29; 0.58)<br>FB only: 0.1 (0.34; 0.54)
Greene *et al*. (2012) (62)	Online education	*Data as mean (SE) F&V cups/day*<br>**Intervention:** 2 item screener: pre: 2.6 (0.10) post: 3.7 (0.10)<br>NCI: pre: 3.3 (0.12), post: 4.1 (0.16)<br>**Control:** 2 item screener: pre: 2.6 (0.09), post: 2.5 (0.10)<br>NCI: pre: 3.1 (0.12), post: 2.8 (0.15)<br>***P*** <0.001 (for both tools)	*F&V* 2 Item<br>0.46 (0.35; 0.57)<br><br>*F&V* NCI<br>0.32 (0.21; 0.43)
Hebden *et al*. (2013) (65)	SMS, Apps, Email, Website	*Data as median serves/day (IQR 25–75%)*<br>**Intervention:** V: pre: 2.0 (1.0–3.0), post: 2.0 (2.0–4.0) F: pre: 1.5 (1.0–2.0), post: 2.0 (1.0–3.0)<br>**Control:** V: pre: 2.0 (2.0–3.0), post: 3.0 (2.0–4.0) F: pre: 2.0 (1.0–2.0), post: 2.0 (1.0–3.0)<br>V: ***P***= 0.66 , F: ***P***= 0.96	*Vegetables*<br>0.12 (-0.43; 0.67)<br>*Fruit*<br>0.01(-0.53; 0.60)
Kattelmann *et al*. (2014) (63)	Online education, Email	*Data as mean (SD) F&V cups/day*<br>**Intervention:** pre: 2.6 (2.1), post: 2.8 (2.1)<br>**Control:** pre: 2.7 (1.9), post: 2.5 (2.1)<br>***P***= 0.001	*F&V*<br>0.14 (0.03; 0.25)
Kothe & Mullan (2014) (68)	Email	*Data as mean (SD) F&V serves/day*<br>**Intervention:** pre: 4.69 (1.92), post: 5.31 (2.08)<br>**Control:** pre: 4.59 (2.22), post: 5.02 (2.10)<br>***P***= 0.499	*F&V*<br>0.14 (-0.2; 0.48)
Kypri and McAnally (2005) (74)	Online assessment, Email	*Data as % meeting F&V recommendations*<br>**Intervention:** pre: 24%, post 33%<br>**Control:** pre: 21%, post 26%<br>***P***= 0.44	*F&V*<br>0.19 (-0.15; 0.52)
LaChausse (2012) (71)	Online web-based education	*Data as mean (SD) frequency of F&V consumption*<br>**Intervention:** F: pre: 2.67 (1.25), post: 3.37 (1.6), V: pre: 2.44 (1.22), post: 2.80 (1.35)<br>**Control:** F: pre: 3.24 (1.55), post: 3.15 (1.48), V: 2.65 (1.32), post: 2.8 (1.35)<br>***P****=0.04*	*Vegetables*<br>0 (-2.35; 2.35)<br>*Fruit*<br>0.14 (-0.12; 0.40)

**Author (year), citation**	**e/mHealth strategies**	**Results**	
		**Baseline to post intervention**	**Effect Size Cohen's d (95% CI)**
Nitzke *et al.* (2007) (64)	Phone calls	*Data as mean (SD) F&V serves/day*[3]<br>**Intervention:** F: pre: 2.36 (2.5), post 2.59 (4.11) V: pre: 1.69 (1.33), post: 1.82 (1.40) (***P*** < 0.05)<br>**Control:** F: pre: 2.37 (2.36), post: 2.21 (1.96), V: pre: 1.67 (121), post: 1.67 (121)<br>COMBINED F&V<br>**Intervention:** pre: 4.04 (3.18), post 4.40 (4.58)<br>**Control:** pre: 4.03 (3.10), post: 3.87 (2.67) (***P*** < 0.05)	*Vegetables:*<br>0.11 (0.03; 0.20)<br>*Fruit:*<br>0.12(0.03; 0.21)<br><br>*F&V*<br>0.14 (0.03; 0.26)
Partridge *et al.* (2015) (66)	SMS<br>Mobile Apps<br>Website<br>Email	*Data as % consuming ≥2 F & ≥4 V serves/day*<br>**Intervention:** F: pre: 33.3%, post: 75.6%, V: pre: 15.4%, post: 35% **Control:** F: pre: 38.4%, post: 60.0%, V: pre: 14.4 %, post: 22.4%<br>F: ***P*=** 0.18, V: ***P***=0.009[4]	*Vegetables*:<br>0.40 (0.04; 0.70)<br>*Fruit:*<br>0.8 (0.33; 1.31)
Richards *et al.* (2006) (75)	Website<br>Email<br>Phone calls	*Data as mean (SE) F&V serves/day*<br>**Intervention:** pre: 2.2 (0.1), post: 3.2 (0.1)<br>**Control:** pre: 2.1 (0.1), post: 2.5 (0.1)<br>***P*** < 0.001	*F&V*<br>0.56 (0.33; 0.79)
Rompotis *et al.* (2014) (72)	SMS<br>Email	**All groups:** Pre: 2.32, post: 2.66 (+0.34 V serves/day across the groups)<br>***P*** < 0.009 (change in intake NR by group or for fruit)<br>NS differences between groups *P*=0.30	N/A<br>No control vs intervention mean and SE/SD for calculation
Shahril *et al.* (2013) (70)	SMS	*Data as mean (SE) F&V serves/day*<br>**Intervention:** F[5]: pre: 0.40 (0.05), post: 1.16 (0.08), V: pre:1.39 (0.06), post: 1.45 (0.06)<br>**Control:** F[5]: 0.35 (0.04), post: 0.32 (0.04), V: pre: 1.31(0.06), post: no change in V<br>F: ***P*** < 0.001, V: ***P***=0.12	*Fruit*<br>1.0 (0.8; 1.2)<br>*Vegetables*<br>0.17 (-0.03; 0.37)

[1], data based on single item F&V tool as no post test data for FFQ; [2], Intervention + feedback group compared to the control; [3] 3 month data reported (6 month NA); [4] p values based on shift in intake for intervention vs. control arms, [5] including fruit juice; BCT, behavior change techniques; FB, feedback, FFQ, food frequency questionnaire; F&V, fruit and vegetables; I, intervention; IQR. Interquartile range; msg, message; NA, not applicable; NCI, national cancer institute; NR, not reported; NS, not significant; PA, physical activity; SMS, short message service; SOC, stages of change; SSB, sugar sweetened beverages; TA, take away; wks, weeks
